# Protocol for a mixed methods feasibility and implementation study of a community-based integrated care model for home-dwelling older adults: The INSPIRE project

**DOI:** 10.1371/journal.pone.0278767

**Published:** 2022-12-21

**Authors:** Olivia Yip, Maria Jose Mendieta, Leah L. Zullig, Andreas Zeller, Sabina De Geest, Mieke Deschodt, Flaka Siqeca, Franziska Zúñiga, Matthias Briel, Matthias Schwenkglenks, Carlos Quinto, Suzanne Dhaini

**Affiliations:** 1 Nursing Science, Department of Public Health, University of Basel, Basel, Switzerland; 2 Academic Center for Nursing and Midwifery, Department of Public Health and Primary Care, KU Leuven, Leuven, Belgium; 3 Department of Population Health Sciences, Duke University School of Medicine, Durham, North Carolina, United States of America; 4 Center of Innovation to Accelerate Discovery and Practice Transformation, Durham Veterans Affairs Health Care System, Durham, North Carolina, United States of America; 5 Centre for Primary Health Care, University of Basel, Basel, Switzerland; 6 Gerontology and Geriatrics, Department of Public Health and Primary Care, KU Leuven, Leuven, Belgium; 7 Competence Center of Nursing, University Hospitals Leuven, Leuven, Belgium; 8 Division of Clinical Epidemiology, Department of Clinical Research, University Hospital Basel and University of Basel, Basel, Switzerland; 9 Department of Health Research Methods, Evidence and Impact, McMaster University, Hamilton, Canada; 10 Department of Public Health, Institute of Pharmaceutical Medicine (ECPM), University of Basel, Basel, Switzerland; 11 Aerztegesellschaft Baselland, Basel, Switzerland; Public Library of Science, UNITED KINGDOM

## Abstract

**Background:**

Evaluations of integrated care models for home-dwelling frail older adults have shown inconclusive results on health and service outcomes. However, limited research has focused on the implementation of integrated care models. Applying implementation science methods may facilitate uptake of integrated care models, thus generating positive outcomes e.g., reduced hospital admissions. This paper describes the protocol to assess the feasibility of an integrated care model (featuring a four-step comprehensive geriatric assessment: screening, a multi-dimensional assessment, a coordinated individualized care plan and follow-up) designed for a new community-based center for home-dwelling older adults in Switzerland. The study includes the following objectives: 1) to assess implementation by a) monitoring respondents to the outreach strategies and describing the Center’s visitors; b) assessing implementation outcomes related to the care model (i.e., adoption, acceptability, feasibility, fidelity) and implementation processes related to collaboration; and 2) assessing implementation costs.

**Methods:**

For objective 1a, we will use a descriptive design to assess respondents to the outreach strategies and describe the Center’s visitors. We will use a parallel convergent mixed methods design for objective 1b. Implementation outcomes data will be collected from meetings with the Center’s staff, interviews with older adults and their informal caregivers, and reviewing older adults’ health records at the Center. Implementation processes related to collaboration will be assessed through a questionnaire to external collaborators (e.g., GPs) towards the end of the study. For objective 2, implementation costs will be calculated using time-driven activity-based costing methods. Data collection is anticipated to occur over approximately six months.

**Discussion:**

This study of a contextually adapted integrated care model will inform adaptations to the outreach strategies, care model and implementation strategies in one community center, prior to evaluating the care model effectiveness and potentially scaling out the intervention.

**Trial registration:**

Feasibility study registration ID with clinicaltrials.gov: NCT05302310; registration ID with BMC: ISRCTN12324618.

## Introduction

Home-dwelling older adults are a growing population, often striving to maintain independence despite their rates of multimorbidity and/or frailty [1], and complex health and social needs [2, 3]. To help meet home-dwelling frail older adults’ complex needs and address problematic care fragmentation, health and social care systems can aim to provide integrated person-centered care [4, 5]. Achieving integrated person-centered care requires actions at the system and service level, and in clinical practice, such as conducting comprehensive assessments of older adults at risk of being frail and forming networks between providers, to help improve service coordination within and between sectors [6]. Despite the strong evolving frameworks and guidance on planning and delivering integrated care for this population [7–9], implementation of integrated care is challenging [10, 11]. It is considered a complex intervention which includes multiple components, targets multiple levels and involves multiple players [11, 12]. While evaluations of integrated care for home-dwelling older adults have demonstrated limited evidence of effectiveness [13–16], these evaluations have rarely assessed the implementation during their evaluation [10, 16, 17], which is a critical gap in integrated care research.

Systematic reviews on integrated care for home-dwelling older adults with multi-morbidity and/or frailty have demonstrated inconclusive results for health and service outcomes (e.g., reduced mortality rates and hospital admissions), and heterogeneity in terms of the intervention components and delivery, and the selected outcomes [14–16, 18–20]. However, these studies rarely assessed implementation processes and outcomes [16]. Kumpunen et al. (2020) proposed three ideas behind why integrated care initiatives often fail, relating to their design (e.g., the model lacks a logical underpinning for the changes expected), delivery (e.g., due to implementation issues or lacking fidelity), or evaluation (e.g., evaluations fail to account for the context) [21]. There are many known barriers to implementing integrated care, including training, leadership, trusting relationships, or unclear roles [11, 21]. Given the common methodological gap in previous integrated care studies, this indicates the need to focus on the implementation of community-based integrated care models to determine whether the negative conclusions were due to intervention failure or implementation failure [16, 22]. Therefore, an implementation science approach can be used, which is “the scientific study of methods to promote the systematic uptake of research findings and other evidence-based practices into routine practice” [23]. This includes key elements such as engaging stakeholders, measuring implementation outcomes (e.g., acceptability and fidelity) and investing in implementation strategies (i.e., strategies to support uptake and implementation of an intervention, such as educational outreach visits [24]) to overcome implementation barriers [10, 16, 17]. Incorporating implementation science principles and methods has the potential to enhance “fit” with real world work flows, facilitate the uptake of integrated care in practice [10, 16, 17, 23, 25], and enhance effectiveness.

In Switzerland, the development and implementation of integrated care initiatives are less advanced than in other European countries [26, 27]. Previous authors suspected this is likely related to 1) the lack of a federal regulatory framework for integrated care and 2) features of the fragmented care system, partially driven by the structure and financing of care, e.g., responsibilities for health care are divided across three levels (federal, cantonal, and local) [26, 27]. This has likely contributed to only smaller integrated care initiatives taking place in different local areas [26, 27]. An additional challenge in Switzerland is that while there are many health and social offers available to older adults, they are not centrally coordinated or easy to navigate [28]. As previously described [22], a care law introduced in 2018 in the Canton Basel-Landschaft (BL) in the north-west of Switzerland, required re-organization of the Cantonal municipalities into nine new care regions [29]. Subsequently, each care region needed to establish an Information and Advice center (IAC) to support older adults with matters related to care and nursing in old age [29]. Our research team has collaborated with the Canton and care regions on a multi-phase implementation science project, the INSPIRE project (ImplemeNtation of a community-baSed care Program for home dwelling senIoR citizEns). INSPIRE aims to develop, implement and evaluate a community-based integrated care model for the IAC. The INSPIRE project includes three phases based on the Medical Research Council framework for developing and evaluating complex interventions [30]. The first phase focused on development and consisted of a systematic literature review and meta-analysis [16], contextual analysis including a population survey [31], and stakeholder involvement to develop a contextually-adapted integrated care model; this body of work has been previously described in detail [22]. Considering the well-known challenges involved with implementing and evaluating integrated care in practice, a feasibility study is crucial to understand how the initial implementation is going and make any necessary adaptations, prior to evaluating its effectiveness. Therefore, building on the completed work from the development phase, during the second phase we assess the feasibility of the contextually developed model. This article is the protocol which describes the methodology for assessing the feasibility of the INSPIRE integrated care model.

The specific objectives of the planned study are to 1) assess the implementation of the integrated care model by: a) monitoring respondents to the outreach strategies and describing the IAC visitors (e.g., number of visitors, reason for appointment); b) assessing implementation outcomes (i.e., adoption, acceptability, feasibility, and fidelity) from the perspective of older adults and their informal caregivers, the IAC nurse and social worker, as well as assess implementation processes related to interprofessional collaboration; and 2) assessing implementation costs (i.e., costs associated with the implementation strategies for the IAC care model [32]). Overall, findings will determine if adaptations are needed to the outreach strategies, care model or the implementation strategies.

## Materials and methods

The methods for this feasibility study are reported according to the StaRI [33, 34] and SPIRIT 2013 guidance [35], to provide transparent and accurate reporting of the protocol for both an intervention and implementation study.

### Study design

For objective 1a, a descriptive design will be used to assess the respondents to the outreach strategies and describe the IAC visitors. To address objective 1b, a parallel convergent mixed methods observational design will be used, integrating data on the care model implementation from quantitative (e.g., IAC administrative data) and qualitative sources (e.g., interviews). For objective 2, a cost analysis will be conducted to determine implementation costs.

### Study context and setting

To understand the context for this intervention, a contextual analysis was performed during the first phase of the INSPIRE research project using the Context and Implementation of Complex Interventions (CICI) framework [36], resulting in a rich description of contextual factors and the implications on the intervention and implementation strategies. This contextual information is reported in detail elsewhere [22].

This study will be conducted in the new community-based IAC in one German-speaking care region of Canton BL, Switzerland. The care region is comprised by a mix of urban and rural areas, and approximately one-tenth of the total population of the care region was aged 75+ in 2020 [37]. The IAC receives public funding from the multiple municipalities that belong to the participating care region. This new center employs a manager, one administrative support, a nurse (currently 0.8 FTE; a geriatric nurse expert; RN and Master’s trained) and a social worker (0.8 FTE).

### Study sample and eligibility criteria

This study is comprised of multiple samples (Table 1):

- All visitors to the IAC
- Older adults: A consecutive sample of older adults will be recruited, and we estimate that a maximum of 18 will participate. Using a purposeful sampling strategy, a nested sample of older adults will be invited for interviews. As analysis will occur in parallel with data collection, we believe information power (a concept proposed by Malterud et al. (2016) which can help inform sample size) could likely be achieved in a minimum of 8 interviews [38].
- Informal caregivers
- IAC staff
- External collaborators

**Table 1:** Eligibility criteria for study samples.

	Inclusion criteria	Exclusion criteria
**Objective 1a**		
**All visitors**	• all individuals who visit/contact/have a home-based appointment with the IAC	
**Objective 1b**		
**Older adults–IAC health record review** (n = 18)	• consenting German- or English-speaking older adults aged 64+ who live at home or are staying temporarily in a nursing home • had a visit at the IAC or at their home • had an IAC health record created	• individuals who are residing in a nursing home or are receiving end-of-life care
**Older adult–interviewees** (n = 8–12)	• individuals with a frailty screening score (using the Groningen Frailty Indicator [GFI]) of ≥4 • receive a Comprehensive Geriatric Assessment (CGA) by the IAC staff • aged 75+ and consent (or by proxy)	• individuals participating in another study with health-related interventions within the 30 days preceding or during the present study
**Informal caregivers** (n = 8–12)	• a participating older adult who was invited for an interview agrees they can be contacted • attendance at the IAC appointment with the older adult	• the older adult did not allow their study invitation • no attendance at the IAC appointment
**IAC staff** (n = 2)	• IAC nurse (i.e., a geriatric nurse expert) and IAC social worker	• IAC administrative support and IAC manager
**External collaborators** (n = 18)	• health or social care providers (e.g., GPs, home care nurses) who according to the IAC health record have worked together with the IAC staff in coordinating care for a participating older adult	• providers who have not contributed to the coordination of care with the IAC staff

**Fig 1:**
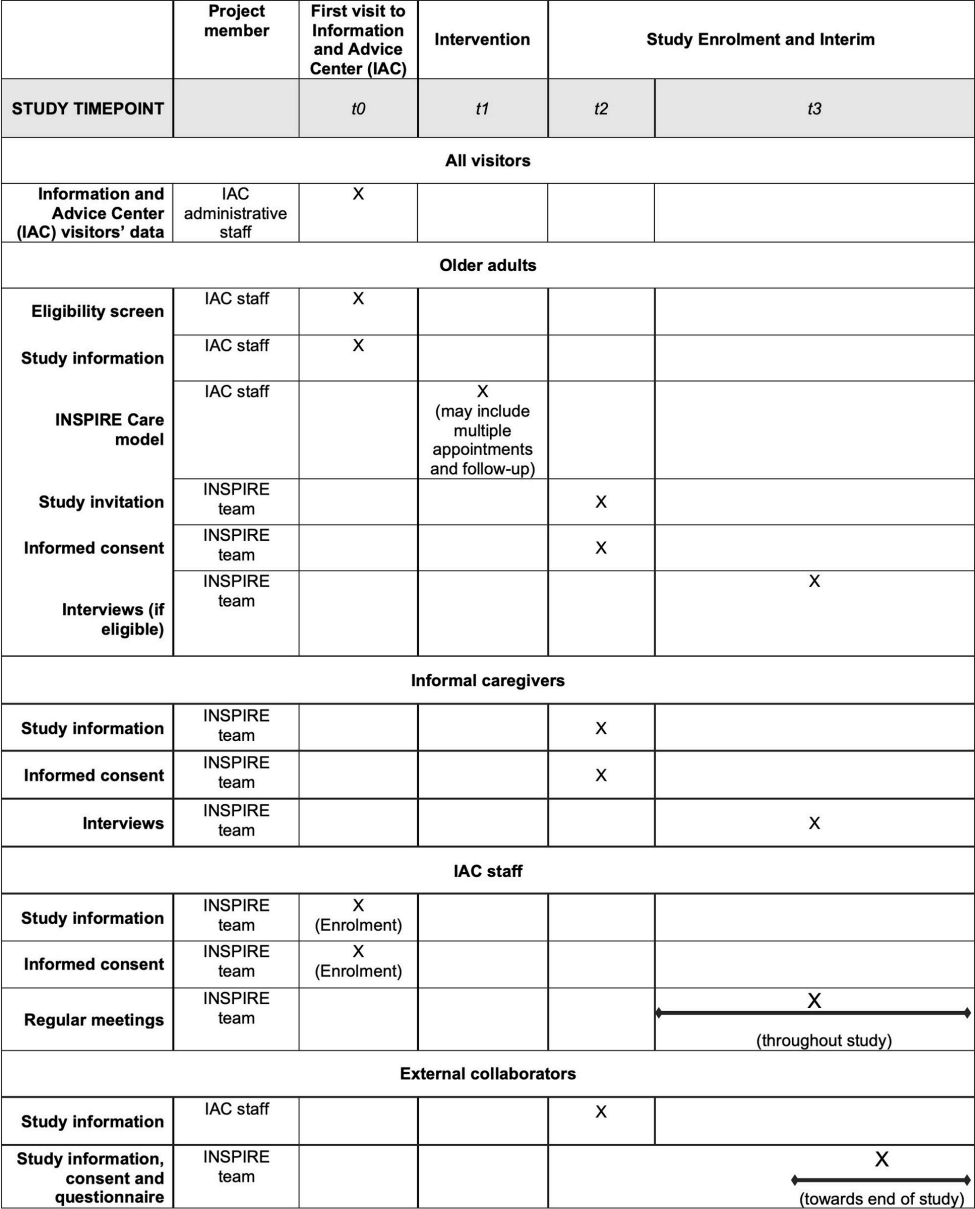
SPIRIT schedule of procedures. IAC = Information and Advice Center. T0 = opening of the new IAC; study enrolment of IAC staff; information collected about all IAC visitors; screening of older adults. T1 = intervention (usual care at the new IAC) which takes place regardless of whether older adults participate in the study. T2 = enrolment of eligible older adults and their informal caregivers as identified in t1. T3 = interviews of participating older adults and informal caregivers; meetings with IAC staff throughout study; survey of external collaborators.

S1 File describes the flow of selected sample sets to collect the study data. The participant timeline is shown in Fig 1.

### Recruitment

**Fig 2:**
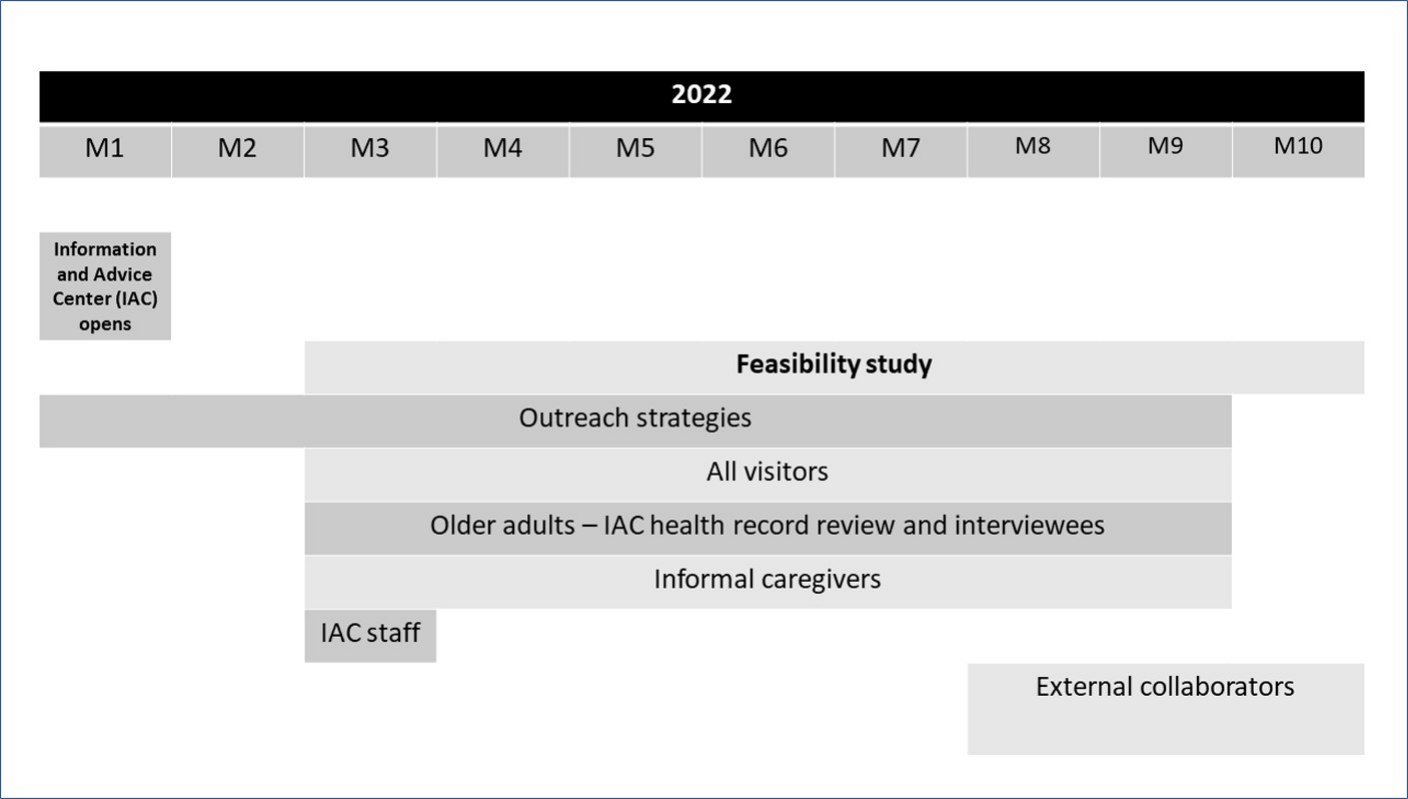
Recruitment timeline. M = Month.

Recruitment for the study will take place on a timeline as shown in Fig 2.

### All visitors

After consultation with stakeholders (i.e., IAC staff, local health and social care organizations/providers as well as other INSPIRE Cantonal stakeholders), and guided by the literature [22], selected outreach strategies will be used to promote the IAC across the community and help support referrals and collaboration with the IAC. Therefore, organizations caring for older adults (e.g., home care), community venues (e.g., churches), healthcare facilities (e.g., hospitals), as well as health and social care professionals (e.g., GPs, home care nurses) will be informed about the IAC purpose and target audience. Outreach strategies will be targeted to specific audiences, e.g., newspaper ads for widespread promotion and outreach meetings with hospitals.

Older adults and their informal caregivers will also be contacted directly through, for example, individual letters by the IAC staff, to raise awareness about the IAC and encourage them to visit the center. Assuming the outreach strategies will lead to individuals contacting/visiting the IAC, the IAC administrative staff will subsequently record data about these visitors.

Recruitment of service users and care professionals will last for around six months.

Service users: Older adults and informal caregivers

The IAC nurse will inform eligible older adults about the study. The research team will contact older adults who are interested in participating in the study, and their informal caregivers to invite them to participate.

Care professionals: IAC nurse and social worker and external collaborators

The IAC staff and external collaborators (identified via IAC health records) will be contacted first by the manager of the IAC, before being invited to participate by the research team.

### The INSPIRE care model–a complex intervention

As a result of the activities in the development phase (i.e., a literature review, contextual analysis, and stakeholder involvement) [22], we developed the integrated care model (i.e., a complex intervention) concept for the IAC and described the underlying program theory using a logic model. Though originally outlined in our development paper [22], the care model features a Comprehensive Geriatric Assessment (CGA) with four “core components”:

- Screening of older people for risk of frailty using a frailty screening tool, the Groningen Frailty Indicator [39]. This tool will be administered at intake to identify the appropriate care required. Older adults with low risk of frailty (GFI < 4; [39]) will receive health promotion, social and financial support, and preventive care from the IAC social worker.
- Older adults at risk of frailty (GFI ≥ 4) will receive the remainder of the CGA including a multi-dimensional assessment, a coordinated individualized care plan and follow-up, all of which will be delivered by the IAC nurse and social worker over two to three appointments (at the older adults’ home or at the IAC, depending on their situation). During the first part of the assessment, the IAC nurse will complete the WHO ICOPE guidance for person-centred assessment and pathways in primary care (ICOPE Handbook) screening tool [7] to identify six “priority conditions associated with declining intrinsic capacity” (i.e., cognitive decline, limited mobility, malnutrition, visual impairment, hearing loss, and depressive symptoms). Any areas that require further assessment as well as additional domains (e.g., sleep and medications) will be assessed. This first section of the multi-dimensional assessment is expected to take between 30 to 60 minutes, depending on the number of assessments required and the older adults’ situation. The IAC social worker will conduct the next part of the multi-dimensional assessment (approximately 30 minutes) in the older person’s home or at the IAC, assessing social care and support (e.g., finances, living environment) following an adapted version of the respective WHO ICOPE care pathway.
- Next, based on the assessment data (including a list of problems), an individualized care plan will be created by the IAC nurse and social worker, and discussed with the older person as well as their informal caregivers and professionals involved in their current care, either in person, by phone or email. Care planning will include understanding the older adult’s needs, goals and preferences (e.g., what is important to them); views of health and social care professionals (i.e., medical history, conditions and priorities); care and support arrangements; and developing an action plan. The IAC staff will coordinate the care plan by having an overview of the older adults’ needs and goals, current care/support, and new care/support recommended; sharing and monitoring the care plan with the older adult, informal caregivers and other care providers to ensure they are meeting the older adults needs; as well as providing interventions and managing referrals to other providers/organizations.
- Lastly, the IAC staff will follow-up on a case-by-case basis, according to: when they agreed to follow-up with the older adult and their informal caregiver when creating the care plan; if referrals are recommended for an external care organization; or if required based on subsequent interactions with their other care providers (e.g., GP or home care nurse). Follow-up includes monitoring progress of the older person and detecting difficulties in participating in interventions, adverse effects of interventions, and changes in functional status. The older adult’s IAC health record will remain open, but the follow-up will cease when no further action is required from the IAC staff according to the older adult’s needs.

For older adults at highest risk and/or who have come to the IAC with a recommendation from a care professional for referral to a nursing home, the IAC staff will collaborate with other professionals involved in their care to determine whether a nursing home referral is needed.

The adaptable “peripheral components” of the care model constitute elements related to the intervention and organization into which it is being implemented, which could be adapted to facilitate the implementation of the care model: place of delivery, tools used for the screening or multidimensional assessment, time spent and number of appointments to deliver the intervention, and staff delivering the intervention [40].

### Implementation strategies

Based on a contextual analysis and review of existing evidence during the development phase, we identified factors which may influence the core components and operationalization of the integrated care model and the strategies needed to ensure successful implementation of the model. Then, we selected and operationalized implementation strategies to support the care model with the input of our operational partners, including IAC staff, health and social care organizations/providers, and political representatives from specific care regions within Canton BL [22]. Consistent with the Expert Recommendations for Implementing Change (ERIC) taxonomy, the strategies included: *use evaluative and iterative strategies; adapt and tailor to context; develop stakeholder interrelationships; train and educate stakeholders; support clinicians;* and *engage consumers* [41], all of which are contextually-adapted and reported in more detail elsewhere [22]. Additional implementation strategies have since been added, which are described along with the expansion of previous key strategies in Table 2.

**Table 2:** Expansion on previous implementation strategies selected for INSPIRE and additional implementation strategies.

ERIC cluster	ERIC Implementation strategy	Description of the implementation strategy in the INSPIRE project
*Expansion of previous strategies*		
**Train and educate stakeholders**	Conduct ongoing training Make training dynamic	The training curriculum (e.g., physiology of ageing and geriatric syndromes, CGA competencies, community health leadership and communication) was carefully designed by the research team for the IAC nurse and social worker. Extra training was given on requested topics such as delirium and dementia. The training program for the IAC nurse and social worker has taken approximately 31 hours to date (as part of the IAC staff’s regular hours) and has included consulting by the research team when needed.
**Engage consumers**	Use mass media	Advertisement material was developed and communicated using different channels to promote awareness of the IAC across the community.
*New strategies*		
**Change infrastructure**	Change record systems	The INSPIRE team will meet with IAC staff to suggest adaptations to the software used in the IAC.
**Train and educate stakeholders**	Provide ongoing consultation	The INSPIRE team will provide ongoing support to the IAC staff (e.g., coaching, telephone calls, and/or supervision).

S2 File describes the research team roles, the roles of the IAC staff who are delivering the care model, and the areas reflecting collaboration.

### Adaptations to the intervention and implementation strategies

Adaptations to the core and peripheral components of the intervention and implementation strategies that occur during the feasibility study could be suggested by either the IAC staff or the INSPIRE research team. Such adaptations to enhance fit can generally help to improve outcomes [42]. The INSPIRE team will be informed about these changes due to regular in-person meetings and consultations with the IAC staff. Adaptations will be proactively tracked by a member of the research team using the updated Framework for Reporting Adaptations and Modifications–Expanded (FRAME) for the core or peripheral intervention adaptations [43], and FRAME for Implementation Strategies (FRAME-IS) for modifications to the implementation strategies [44].

### Variables and measurement

Table 3 summarizes all variables to be collected during the feasibility study and Table 4 describes the baseline characteristics of all visitors and consenting older adults.

**Table 3:** Summary of feasibility study variables and data sources.

Outcome variables	Variable description/definition	Data source (and data type^1^^,^^2^)	Data collection timeframe
Objective 1a: monitor respondents to the outreach strategies and describe IAC visitors			
Respondents to outreach strategies	The proportion of individuals/organizations who respond to each outreach strategy used to promote the IAC (e.g., % of GPs who received the promotional video about the IAC and subsequently referred older adults or % of hospitals who referred older adults to the IAC after an outreach meeting)	• IAC administrative data^1^ • IAC administrative data^1^	• Weekly
Number of visitors and appointments	The total number of visitors to the IAC or home-based appointments, and the number of appointments		
Reason for appointment/ contacting the IAC and referral source	The primary reason for contacting the IAC/booking an appointment and the source of referral for each older adult who visits the IAC		
Type of service received by visitors	The number of older adults who during their IAC appointment receive either a) health promotion and prevention as well as social and financial support; b) a full CGA; c) a brief assessment to confirm whether a nursing home referral is warranted; or d) other		
Objective 1b: assess the implementation outcomes and processes			
*Implementation outcomes (i.e., essential proximal outcomes to assess within the overall system before we can measure effectiveness outcomes [43])*			
Adoption	The intention or action of the IAC staff to employ the care model [45]*, which will be determined through the researchers’ perceptions during the meetings with IAC staff and the staffs’ documentation in the IAC health record.	• IAC staff meeting log^2^ • IAC health record^1^	• Regular meetings will be held with the IAC staff
Acceptability	The perception among IAC staff and service users that the care model is agreeable [45]*.	• IAC staff meeting log^2^ • Older adults & informal caregiver interviews^2^	• Regular meetings will be held with the IAC staff • Interviews will be held within two-weeks of second appointment
Feasibility	The extent to which the care model can be successfully implemented within the IAC of the care region [45]*. Feasibility will be assessed for each component of the care model and overall.	• IAC staff meeting log^2^ • Older adults & informal caregiver interviews^2^	
Fidelity	The degree to which the core components of the care model is implemented according to protocol [45]*, largely based on the assessments conducted.	• IAC health record^1^ • IAC staff meeting log^2^	• Weekly and at the end of the study
*Implementation processes*			
Implementation processes–collaboration	The implementation processes related to collaboration between IAC staff and external health and social care professionals to coordinate care for an older adult [46]. This will be captured from the perspective of the external professionals who collaborate with the IAC staff.	• The Normalization MeAsure Development questionnaire (NoMAD ^1^) [46]	• Towards the end of the study
Objective 2: assess implementation costs			
Implementation costs (i.e., costs related to the implementation strategies)	The time-driven activity-based cost related to the planning and delivery of implementation strategies [32]	• IAC administrative data^1^ and INSPIRE document-ation^1^	• Daily

^1^ Quantitative;

^2^ Qualitative;

* Definitions adapted from Proctor et al. (2011)

**Table 4:** Baseline characteristics of all visitors and consenting older adults.

Variables	Variable description/definition	Data source	Data collection timeframe
*All visitors*			
Visitors’ sociodemographics	• Age, gender, municipality of residence	• IAC administrative data	• Weekly
*Individual baseline characteristics of consenting older adults*			
Demographic data	• Year of birth, gender, education, number of people living in the same household, household constituents	• IAC health record	• Weekly
Geriatric risk profile	• via the Groningen Frailty Indicator [39]		
Cognition	• via the ICOPE Screening questions [7] and mini-cog [47]		
Depressive symptoms	• via the ICOPE screening questions and mood assessment [7]		
Multimorbidity	• the occurrence of two or more chronic diseases [48] • determined by their number of chronic illnesses/conditions		
Nutritional status	• via the ICOPE screening questions and Mini Nutritional Assessment–Short Form [49, 50]		
Fall history	• assessed by asking if they had two or more falls in the previous 12-month period [51]		

### Data collection, management and analysis

#### Data collection methods

For objective 1a, summarized IAC administrative data will be provided to the research team to monitor/assess the respondents to the outreach strategies used to promote the IAC and describe the IAC visitors.

For objective 1b, the IAC health records of participating older adults will be reviewed by the research team using the investigator-created fidelity tool to assess fidelity (Supplementary file 3). The individual sample characteristics of the older adults will also be captured weekly from the IAC health records to describe this sample. Meetings will be held with the IAC staff to contribute information on fidelity. To assess acceptability and feasibility, the research team will individually interview eligible older adults, i.e., “interviewees”, and their informal caregivers (separately) during 45-minute semi-structured interviews. Meetings will also be held with the IAC staff to explore their perspective on acceptability and feasibility, as well as adoption. To assess the IAC implementation processes regarding the collaboration with external professionals for care coordination, external collaborators will be asked to complete the NoMAD questionnaire [46].

For objective 2 (to calculate implementation costs), both the INSPIRE research team and the IAC staff will record the amount of time invested in designing and delivering implementation strategies. For example, this includes developing flyers and the training curriculum; training and consulting between INSPIRE and the IAC staff; and conducting meetings with community providers. Data will then be extracted from the IAC administrative data and the research team’s documentation on frequency of activities, time spent per activity per actor, and non-personnel resources invested such as material required or travel costs.

#### Data management

Quantitative study data on older adults, informal caregivers, and IAC staff will be securely stored in Castor Electronic Data Capture (EDC). In Castor, an electronic case report form (eCRF) will be created for each enrolled older adult. The older adult will be assigned a unique code to ensure the data is pseudonymized. The paper-based Master file containing the codes assigned to participating older adults will be securely stored in a locked cabinet in the Institute of Nursing Science for 10 years, separate from the project data. Any paper-based copies of the anonymous NoMAD questionnaire will be stored in a locked cabinet at the Institute of Nursing Science while electronic copies will be downloaded from LimeSurvey and saved on the secure servers of the University in a confidential folder of the research team.

For qualitative data, audio recordings and paper-based notes from all interviews and meeting logs will be stored in the archive in a locked room and cabinet in the Institute of Nursing Science at the University of Basel for 10 years.

To promote data quality, multiple documents were pilot tested with their respective target audience to ensure comprehension, flow and relevance, e.g., the study recruitment letter, the interview guide for older adults, the interview guide for informal caregivers, and the NoMAD survey. Data collectors were trained on appropriate procedures regarding consent, confidentiality, techniques for conducting interviews, and the fidelity tool. A random check of ten percent of the Castor records will take place at the end of the study to ensure data completeness. Trial rounds were held for collecting data from the IAC health record and NoMAD survey.

#### Data analysis

For objective 1a, we will calculate descriptive statistics to describe the respondents to the outreach strategies and the IAC visitors. For objective 1b, descriptive statistics (e.g., mean and SD, median and IQR) and frequencies will be calculated and reported to describe participant demographics, study fidelity and survey results. Our research team will use rapid qualitative analysis as described by Hamilton [52], to analyze qualitative data from the interviews and meeting logs. Two INSPIRE researchers will participate in both the interviews and meetings and take notes independently on a pre-structured meeting log consistent with the components of the INSPIRE care model. These notes will be summarized on a template which was prepared *a priori* with the main topics (i.e., implementation outcomes) [52]. The notes will be coded, analyzed for themes, and further analyzed using a matrix analysis [52]. The audio recordings will be checked should further verification or transcription be needed. The main actionable findings will be shared to guide further implementation in real time.

We will collect and analyze quantitative and qualitative data in parallel before the quantitative and qualitative results will be merged through side-by-side comparisons in joint display tables or in discussions. Similarities and differences will be described between data types.

For objective 2, time-driven activity-based costing methods will be used to calculate implementation costs, based on estimations of activity frequency, time invested per job category, and non-personnel resources invested [32]. All quantitative analysis will be conducted using R program version 4.0.2 [53] and qualitative analysis using nVIVO software version 1.7 [54].

#### Ethical considerations and informed consent

The feasibility study was submitted to the Ethikkommission Nordwest- und Zentralschweiz (EKNZ) in Switzerland, EKNZ 2021–02430. While we were informed on December 14, 2021 that the study did not require a review according to HFG Art. 2, para. 1. under the HRA, the study was re-submitted for an Advisory Opinion and was able to proceed based on positive opinion, as per the EKNZ response on March 3, 2022. The study was registered in ClinicalTrials.gov: NCT05302310.

Written informed consent is sought from the research team via signature from all study participants (except external collaborators whose consent is implied by survey completion).

The nurse will assess cognition of each older adult as part of the regular standard of care during the CGA. If the nurse is concerned about the older adults’ capacity to consent based on the Mini-Cog assessment and their clinical judgement, a proxy consent will be sought (i.e., legal representative). In this case, when the nurse introduces the study to the older adult, the nurse will also ask if the INSPIRE research team can reach out to their legal representative. The INSPIRE research team will speak to the family member/legal representative and older adult, and will ask for proxy consent.

#### Project status

Data collection for the feasibility study will be from March–October 2022. Once analysis of the feasibility data is complete and any adaptations are made, an effectiveness study will begin.

## Discussion

We present the protocol for the feasibility study of an integrated care model for home-based older adults, which will assess the implementation of the integrated care model with respect to respondents to the outreach strategies used and the IAC visitors; assessing implementation outcomes for the care model and implementation processes related to interprofessional collaboration; as well as implementation costs. Given the challenges associated with implementing integrated care, this study aims to fill an important gap of understanding how it could be feasibly and successfully implemented in the community [10, 25, 55, 56], before we can contribute much-needed knowledge about whether integrated care for frail older adults is effective in practice [14]. By assessing the feasibility first, this study gives us an important opportunity to adapt the care model content and how its delivered as well as the implementation strategies.

With this novel study, we bring unique methodological attributes to increase its chances for success and help us to identify any implementation issues prior to measuring the effectiveness outcomes. Of primary importance is the incorporation of implementation science methods, which focus on *how* to implement integrated care [10, 25, 55, 56], and increase the potential for the research findings to be usable in the real world [57]. At its foundation, this began with heavy efforts invested into the development phase to ensure the care model and preliminary implementation strategies were evidence-informed, contextually adapted, and that there is clear logic underlying the program [22]. To assess feasibility of the integrated care model, the current study incorporates key elements of implementation science such as measuring implementation processes and outcomes; using implementation strategies; and stakeholder involvement [58]. For example, given that integration processes are accepted as complex and fairly demanding on stakeholders and professionals [59], it is crucial to use strategies learnt from previous research such as building a multidisciplinary team culture, and shaping roles and competencies for integrated care [56].

The contextually adapted integrated care model under study has the potential to generate positive outcomes on a patient-, provider- and system-level. However, conducting this feasibility study first will determine important preliminary outcomes, such as whether the care model is adopted, acceptable by service users and professionals, and delivered with high-fidelity in practice, before measuring the effectiveness outcomes [45]. Additionally, it is essential to ensure the outreach strategies are driving visitors to the IAC, as it is a new facility. Furthermore, assessing these outreach strategies can give us a better understanding about which strategies enhanced recruitment of home-dwelling frail older adults to visit the Center, which may also benefit future researchers. Collaboration across the community is essential for both referral to the IAC and coordination of care within the care model, hence the importance of capturing the implementation processes related to collaboration from the perspective of external professionals. The combination of these study findings will inform adaptations needed for the outreach strategies, the care model (including both its contents and how it will be delivered), and the implementation strategies.

At the political level, study findings will be beneficial for the local policy-makers who introduced the APG care law, and may help determine how to re-allocate resources or where more policy-related support could help facilitate implementation of integrated care. Given the heavy use of implementation strategies during the feasibility phase, costs associated with this implementation will be especially relevant to local policy-makers (as well as any other Cantons/care regions interested in the care model) as this contributes to the overall costs of running the IAC, and in light of their investment in the IAC. Study findings will be shared with local stakeholders for immediate use as well as broadly disseminated through reports and presentations, academic products, and the INSPIRE website and social media account.

A few potential study limitations should be noted. First, the COVID-19 pandemic adds unpredictable variability to the study as pandemic measures could impact whether the center must experience any temporary closures, older adults’ comfort with visiting the IAC, and the IAC’s ability to collaborate with other professionals. Second, study recruitment may be challenging given one of our samples includes older adults experiencing frailty, and this is a new center in the community, hence our attention to the outreach strategies and respondents. Third, our focus was on creating an intervention and implementation approach with sound contextual fit. As such, the adapted version is well suited for the context. While the core components should be an appropriate blueprint locally and beyond, further adaptation would be needed to either generalize or “fit” the intervention elsewhere. Fourth, the collaboration with, and execution by, care professionals in the community instead of solely researchers presents as both a strength and limitation, as there is more potential for sustainability but also less researcher-control. Lastly, the study timing is impacted by political processes (e.g., the study could not begin before the care regions formed). Nevertheless, the pragmatic “real-world” nature of this study and the methodology used which is grounded in implementation science, remain major strengths.

In conclusion, this is among the limited projects to our knowledge which develop, implement and evaluate an integrated care model for home-dwelling frail older adults using implementation science methods to increase its chance for success. The present feasibility study is an important prerequisite to understand the implementation process and determine if any adaptations are needed before evaluating effectiveness [12] and exploring sustainability.

## Supporting information

S1 File Feasibility study samples, data sources, and outcomes. (DOCX)

S2 File Describing the roles/activities of the IAC staff and the INSPIRE research team during the feasibility study. (DOCX)

S3 File INSPIRE fidelity tool. (DOCX)

S4 File (PDF)
