# Supplementary figures and images for: Protocol for a mixed methods feasibility and implementation study of a community-based integrated care model for home-dwelling older adults: The INSPIRE project
Source: PLoS One. 2022 Dec 21;17(12):e0278767. doi: 10.1371/journal.pone.0278767 (PMC9770388; doi:10.1371/journal.pone.0278767)

**Supplemental File 1.** **Feasibility study samples, data sources, and outcomes**


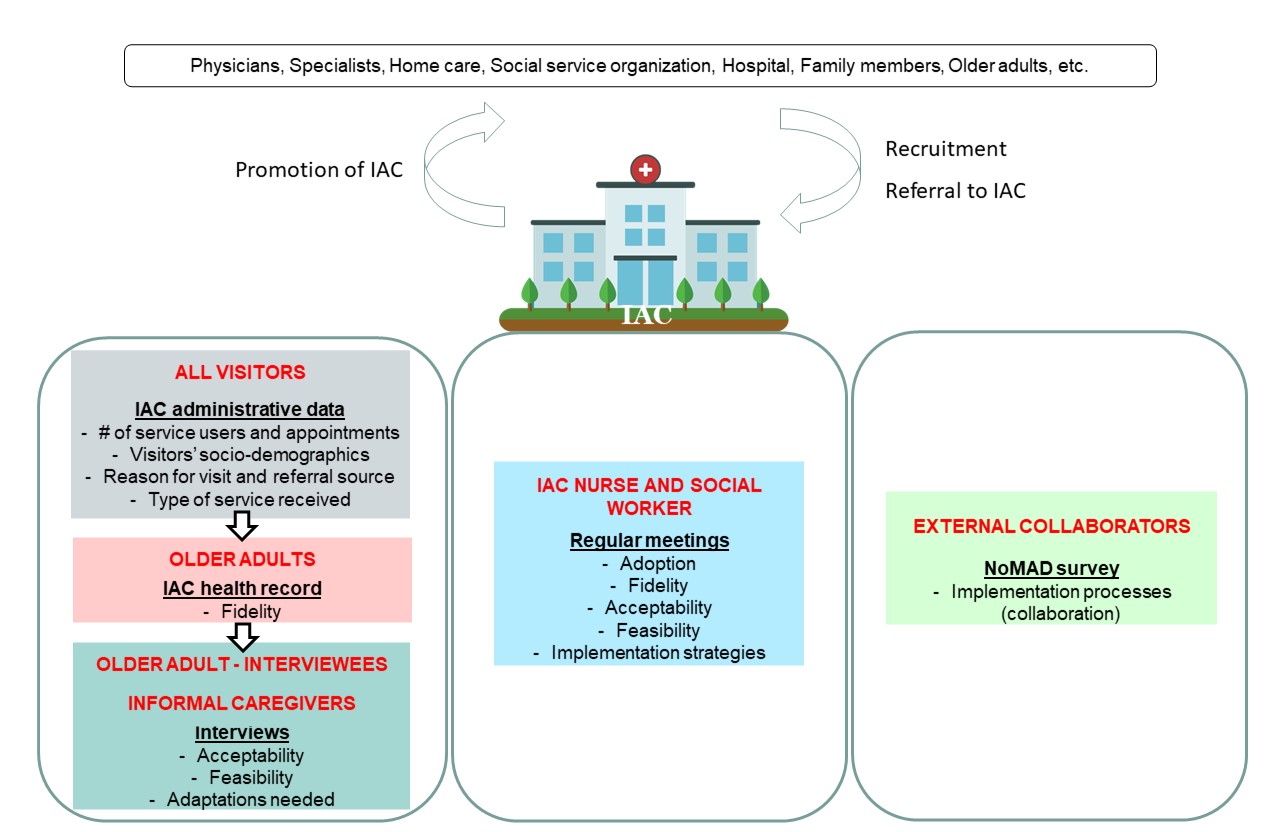

Supplement: S1 File — (DOCX) [file pone.0278767.s001.docx]
