# Supplementary material for: Protocol for a mixed methods feasibility and implementation study of a community-based integrated care model for home-dwelling older adults: The INSPIRE project
Source: PLoS One. 2022 Dec 21;17(12):e0278767. doi: 10.1371/journal.pone.0278767 (PMC9770388; doi:10.1371/journal.pone.0278767)
Supplement: S2 File — (DOCX) [file pone.0278767.s002.docx]

**Supplemental File 2. Describing the roles/activities of the IAC staff and the INSPIRE research team during the feasibility study**

| **IAC Roles** |  | **INSPIRE Roles** |
| --- | --- | --- |
| **Research roles** | | |
| 1A. informs eligible older adults about the study  1B. clarifies IAC health record information as needed | 🡨🡪 | 1. collects data from IAC health records |
|  |  | 2. conducts interviews with older adults and informal caregivers |
| 2. participates in meetings with INSPIRE staff |  | 3. organizes meetings with IAC nurse and social worker |
|  |  | 4. analyzes data and prepares report |
| 3. collaborates with external health and social professionals for care planning |  | 5. develops and administers NoMAD survey to external professionals |
|  |  | 6. manages consent process for all participants |
| **Implementation roles** | | |
| 1. provides IAC services (e.g., provides health promotion and prevention; conducts CGA; nursing home referral) to home-dwelling older adults and provides input on documents | 🡨🡪 | 1. creates process flow, develops educational materials, shapes roles and competencies, conducts ongoing training, organizes meetings with IAC staff |
| 2. conducts outreach strategies to promote the IAC, provides input, and distributes educational materials | 🡨🡪 | 2. develops educational materials for recruitment strategies and assesses recruitment feasibility |
|  |  | 3. tailor strategies |

**🡨🡪** denotes partnership or collaboration in planning/execution
