# Supplementary material for: Protocol for a mixed methods feasibility and implementation study of a community-based integrated care model for home-dwelling older adults: The INSPIRE project
Source: PLoS One. 2022 Dec 21;17(12):e0278767. doi: 10.1371/journal.pone.0278767 (PMC9770388; doi:10.1371/journal.pone.0278767)
Supplement: S3 File — (DOCX) [file pone.0278767.s003.docx]

**Supplemental File 3. INSPIRE Fidelity Tool**

Purpose of tool: to determine the degree to which the intervention was implemented as it was planned in the original protocol. Fidelity will be measured with this tool using two data sources: 1) reviewing patients’ Fachstelle health record; 2) nurse and social worker’s other notes to determine if the intervention was delivered as intended. The tool below describes the quantitative fidelity items.

- Types of files to be reviewed = INSPIRE study participants only (sample 1B)
- # of files to be reviewed in feasibility study = consecutive sample of all eligible participants
- Individual responsible for data entry = INSPIRE research team
- Data entry schedule = every week and once at the end of the feasibility study to review completion of follow-up activities
- Scoring is done by tallying up the (1)’s at the end to calculate the percentage of the number of cases in which specific component was scored with “yes”/1 over the total number of Fachstelle health records (FHR) reviewed.
- Comments section is for analysts to track any questions which may need clarification from Fachstelle staff or any important details (e.g., if the CGA had to be paused because patient wasn’t feeling well)

**1. Was there a CGA for this client?  YES NO**

**If no, what type of service was completed for this client?**

**Health promotion and prevention Nursing home referral  Other: ___________**

- **Stop data collection.**

**If yes, please complete the tool below.**

**Participant Code: ___________**

|  | ***Data Source*** | **YES** | **NO** | **N/A** | **Comments** |
| --- | --- | --- | --- | --- | --- |
| **SCREENING AND DOCUMENTATION** | | | | | |
| 1A. Was the GFI completed in the Fachstelle health record (FHR)? | *FHR* | YES (1) | NO |  |  |
| 1B. Was a FHR created for each older adult seen by the nurse? | *FHR*  Fachstelle tracking of clients | YES (1) | NO |  |  |
| **COMPREHENSIVE GERIATRIC ASSESSMENT** | | | | | |
| 2A. Is the CGA section in the FHR completed by both the nurse and the social worker? | *FHR*  *Definition of CGA completed: if the screening questions and further assessments are completed by the nurse and social assessment completed by the social worker during their first 1-3 appointments with the Fachstelle* | YES (1) | NO |  |  |
| 2B. Which sections of the CGA were completed? | *FHR*  *Assessment is considered “performed” if the questions were asked for each section.* |  |  |  |  |
| *Screening questions -* *Priority conditions associated with declines in intrinsic capacity* | | | | | |
| - Were screening questions completed on cognitive decline? |  | YES (1) | NO |  |  |
| - Was further assessment of cognitive decline completed? |  | YES (1) | NO | N/A  If no further assessment was required based on screening results |  |
| - Were screening questions completed on limited mobility? |  | YES (1) | NO |  |  |
| - Was further assessment of limited mobility completed? |  | YES (1) | NO | N/A  If no further assessment was required based on screening results |  |
| - Were screening questions completed on malnutrition? |  | YES (1) | NO |  |  |
| - Was further assessment of malnutrition completed? |  | YES (1) | NO | N/A  If no further assessment was required based on screening results |  |
| - Were screening questions completed on visual impairment? |  | YES (1) | NO |  |  |
| - Was further assessment of visual impairment completed? |  | YES (1) | NO | N/A  If no further assessment was required based on screening results |  |
| - Were screening questions completed on hearing loss? |  | YES (1) | NO |  |  |
| - Was further assessment of hearing loss completed? |  | YES (1) | NO | N/A  If no further assessment was required based on screening results |  |
| - Were screening questions completed on depressive symptoms? |  | YES (1) | NO |  |  |
| - Was further assessment of mood completed? |  | YES (1) | NO | N/A  If no further assessment was required based on screening results |  |
| - Were screening questions of delirium completed? |  | YES (1) | NO |  |  |
| - Was further assessment of delirium completed? |  | YES (1) | NO | N/A  If no further assessment was required based on screening results |  |
| - Were screening questions of incontinence completed? |  | YES (1) | NO |  |  |
| - Was further assessment of incontinence completed? |  | YES (1) | NO | N/A  If no further assessment was required based on screening results |  |
| *Assessment questions – Additional health assessment* | | | | | |
| - Was an assessment of sleep performed? |  | YES (1) | NO |  |  |
| - Was an assessment of activities of daily living performed? |  | YES (1) | NO |  |  |
| - Was an assessment of physical activity performed? |  | YES(1) | NO |  |  |
| - Was an assessment of fall risk and history performed? |  | YES(1) | NO |  |  |
| - Was an assessment of pain performed? |  | YES (1) | NO |  |  |
| - Was multimorbidity asked? |  | YES (1) | NO |  |  |
| - Were medications reviewed and analyzed according to the PRISCUS list criteria? |  | YES (1) | NO |  |  |
| - Was the GP contacted to get additional information on health history and medications? |  | YES (1) | NO |  |  |
| *Assessment questions – Social Care and Support* | | | | | |
| - Was the support system and caregivers evaluated? |  | YES (1) | NO | N/A  No assessment with social worker |  |
| - Was an assessment of living conditions completed? |  | YES (1) | NO | N/A  No assessment with social worker |  |
| - Was an assessment of housing conditions completed? |  | YES (1) | NO | N/A  No assessment with social worker |  |
| - Was an assessment of administrative concerns completed? |  | YES (1) | NO | N/A  No assessment with social worker |  |
| - Was an assessment of financial concerns completed? |  | YES (1) | NO | N/A  No assessment with social worker |  |
| - Was an assessment of pets completed? |  | YES (1) | NO | N/A  No assessment with social worker |  |
| - Was an assessment of loneliness completed? |  | YES (1) | NO | N/A  No assessment with social worker |  |
| - Was an assessment of leisure interests completed? |  | YES (1) | NO | N/A  No assessment with social worker |  |
| - Was an assessment of elder abuse risk completed? |  | YES (1) | NO | N/A  No assessment with social worker |  |
| - Was an assessment of spirituality needs completed? |  | YES (1) | NO | N/A  No assessment with social worker |  |
| - Was the older person asked if they have a patient will? |  | YES (1) | NO | N/A  No assessment with social worker |  |
| **3. CARE PLANNING, INTERPROFESSIONAL COLLABORATION AND PATIENT/FAMILY INVOLVEMENT** | | | | | |
| 3A. Was a care plan created in the Fachstelle health record? | *FHR* | YES (1)  *Care Plan is considered created if the problems have been identified and the list of actions have been discussed with the patient (and caregiver) and discussed/shared with the GP within two weeks after the CGA was finished.* | NO | N/A |  |
| 3B. Have the Fachstelle Nurse and Social Worker met at least once to discuss the care plan (within 10-working days of the CGA being completed)? | *FHR*  *Nurse and social worker’s records* | YES (1) | NO | N/A  The patient didn’t see the social worker |  |
| 3C. Have the Fachstelle nurse/social worker shared or discussed the assessment or care plan with other relevant health and social professionals if they were part of the patients’ current care (within 5 working days)?  (Eg health professionals, social support services, other support services (housing, meals, transportation) | *FHR*  *Nurse and social worker’s records* | YES (1) | NO |  |  |
| 3D. Is there clear documentation of the goals of the older adult that has a care plan? | *FHR* | YES (1) | NO |  |  |
| **4. REFERRAL** |  |  |  |  |  |
| 4a. In an older adult identified as needing referral to another health or social professional/service during the CGA, were referral suggestions included in the care plan? | *FHR* | YES (1) | NO | N/A  *No referral was needed* |  |
| 4b. In an older adult identified as needing referral to another health or social professional/service during the CGA, were any referrals arranged? | *FHR* | YES (1) | No | N/A  *No referral was needed* |  |
| **5. FOLLOW-UP AND COORDINATION** |  |  |  |  |  |
| 5a. If the older person needed a follow-up appointment with Fachstelle staff according to the care plan, was the appointment scheduled? | *FHR* | YES (1) | *NO* | N/A  *No follow-up was needed* |  |
| 5b. If the older person needed a follow-up appointment according to the care plan, did the follow-up appointment take place in the time frame defined for the older person? | *FHR* | YES (1) | NO | N/A  *No follow-up was needed* |  |
| 5c. Did the Fachstelle nurse or social worker follow-up with an older person if there is any indication that their condition has changed (e.g., a report of hospitalization, a letter from GP, phone call from family?) | *FHR* | YES (1) | NO | N/A  *No indication that the patient’s condition had changed* |  |
| **6. INTERVENTIONS** |  |  |  |  |  |
| 6a. If the nurse detects problems during the CGA, are nursing actions (e.g., health counselling and prevention) taken as per the protocol? Examples include: | *FHR* | (1) IF EITHER OF THE BELOW ARE COMPLETED |  |  |  |
| - Providing teaching and advice/recommendations when appropriate |  | YES | YES | N/A |  |
| - Providing support and resources when needed |  | YES | NO | N/A |  |

**Total score = ___ / 48 (or total score reduced by “1” for each N/A selected)**
