# Supplementary material for: Protocol for a mixed methods feasibility and implementation study of a community-based integrated care model for home-dwelling older adults: The INSPIRE project
Source: PLoS One. 2022 Dec 21;17(12):e0278767. doi: 10.1371/journal.pone.0278767 (PMC9770388; doi:10.1371/journal.pone.0278767)
Supplement: S4 File — (PDF) [file pone.0278767.s004.pdf]

INSPIRE: Feasibility of a community-based integrated care model for older adults living at home

Research legislation:	Ordinance on human research with the exception of Clinical trials (HRO) (2).
Type of Research Project:	Research project involving human subjects
Risk Categorisation:	Risk A
Project Leader:	Prof. Dr. Sabina De Geest<br>Institute of Nursing Science<br>Departementsleiterin Department of Public Health (DPH)<br>Universität Basel<br>Bernoullistrasse 28, 4056 Basel, Schweiz<br>sabina.degeest@unibas.ch<br>+41 61 207 09 16

PROTOCOL SIGNATURE FORM

Study Title	Feasibility of a community-based integrated care model for older adults living at home

The project leader has approved the protocol version ***[1.0 (dated 14.12.2021)]***, and confirms hereby to conduct the project according to the protocol, the Swiss legal requirements [1, 2], current version of the World Medical Association Declaration of Helsinki [3] and the principles and procedures for integrity in scientific research involving human beings.

Project leader:

Basel

Name: Prof. Dr. Sabina De Geest

Date: 01.03.2022________________ Signature:

TABLE OF CONTENTS

TABLE OF CONTENTS 3
GLOSSARY OF ABBREVATIONS 4
1 BACKGROUND AND PROJECT RATIONALE 5
1.1 From fragmented care to integrated care for home-dwelling older adults 5
1.2 Canton Basel-Landschaft, a test field for Switzerland and Europe 6
2 PROJECT OBJECTIVES AND DESIGN 9
2.1 Hypothesis and primary objective 9
2.2 Primary and secondary endpoints 9
2.3 Project design 13
3 PROJECT POPULATION AND STUDY PROCEDURES 14
3.1 Project population, inclusion and exclusion criteria 14
3.2 Recruitment, screening and informed consent procedure 16
3.3 Study procedures 18
3.4 Withdrawal and discontinuation 20
4 STATISTICS AND METHODOLOGY 20
4.1. Statistical analysis plan 20
4.2. Handling of missing data 21
5 REGULATORY ASPECTS AND SAFETY 21
5.1 Local regulations / Declaration of Helsinki 21
5.2 Notification of safety and protective measures (HRO Art. 20) 21
5.3 Serious events (HRO Art. 21) 21
5.4 Procedure for investigations involving radiation sources 22
5.5 Amendments 22
5.6 End of project 22
5.7 Insurance 22
6 FURTHER ASPECTS 22
6.1 Overall ethical considerations 22
6.2 Risk-Benefit Assessment 23
6.3 *If applicable:* Rationale for the inclusion of vulnerable participants 23
7 QUALITY CONTROL AND DATA PROTECTION 23
7.1 Quality measures 23
7.2 Data recording and source data 24
7.3 Confidentiality and coding 24
7.4 Retention and destruction of study data and biological material 25
8 FUNDING / PUBLICATION / DECLARATION OF INTEREST 26
9 REFERENCES 26

GLOSSARY OF ABBREVATIONS

BL	*Basel-Landschaft*
CGA	*Comprehensive Geriatric Assessment*
EU	*European Union*
GFI	*Groningen Frailty Indicator*
IBS	*Informations- und Beratungsstelle*
INSPIRE	*Implementation of an integrated community-based care program for home-dwelling senior citizens*
LTC	*Long-term care*
MNA-SF	*Mini Nutritional Assessment – short form*
NoMAD	*Normalisation MeAsure Development*
WHO	*World Health Organization*

1 BACKGROUND AND PROJECT RATIONALE

Due to longer life expectancy and lower birth rates, many developed countries currently face an ageing population and an increasing old-age dependency ratio. The EU predicts that by 2060, between 22 and 36% of all citizens will be ≥ 65 years, with 12% of EU citizens being ≥ 80 years (3). In Switzerland, 18.8% of the population was ≥ 65 years in 2020, and this proportion is expected to increase up to 28% in 2060 (4). Although gains in life expectancy are a positive outcome, not all newly-won years will be spent in good health and free of physical limitations. In the 2012 Swiss Health Survey, 53% of the people over 80 were restricted for at least six months in their daily activities due to a health problem, and 45% of the people over 80 reported not to be in good health (5). Because older adults are increasingly faced with disability, multimorbidity and chronic illness, such as diabetes, hypertension, and dementia (6), as well as social isolation and loneliness (7), their needs have evolved from acute to chronic care needs. Hence, the number of home-dwelling individuals depending on health care and social services will continue to increase.

The majority of older people prefer to remain in their homes and community for as long as possible (8), i.e., aging in place, which is defined as "remaining living in the community, with some level of independence, rather than in residential care" (9). However, approximately one third of home-dwelling older people are at risk for not being able to age in place due to functional limitations (10). Ageing in place is not only favoured by many older people themselves, but also by policy makers and health providers, because it avoids the costly option of institutional care (11).

1.1 From fragmented care to integrated care for home-dwelling older adults

The care of older people, often suffering from multiple chronic health problems, disability, frailty, and/or unmet social needs (12), is complex. As a result, many home-dwelling older people receive long-term care by a large number of care providers often in various care settings. Because care across these providers and settings is often neither centralized nor coordinated, older people are at risk for fragmented care (13). Fragmentation of care is characterized by duplication of services, gaps in information delivery, and inappropriate or conflicting care recommendations and inevitably leads to medication errors, confusion and distress for older people and their caregivers, but also to higher care costs due to unnecessary hospitalizations and other unnecessary use of services (14). To address the complex needs of the older population and overcome fragmentation of care, implementation of integrated care models has been recommended by the World Health Organisation (WHO), the National Institute for Health and Care Excellence and the King's Fund, among others (15,16). Integrated care has been described as a person-centred model of care that is structured to support coordinated, pro-active care led by a multidisciplinary core team and a lead coordinator communicating and cooperating across and within health and social sectors (15). Integrated care interventions are complex interventions, with multiple interacting elements (i.e. different health and social care providers) and multiple levels targeted (i.e. organisational level or patient-level) (17). In 2017, Leijten et al. published a literature-based taxonomy of core concepts of integrated care models that were considered relevant to provide integrated care to a multi-morbid population. This resulted in the Sustainable intEgrated chronic care modeLs for multi-morbidity: delivery, FInancing, and performancE (SELFIE) framework (18). Each core concept was categorised in micro-, meso- and macro-level for each of the six domains suggested by the WHO for a well-functioning health system, i.e., delivery of services; leadership; workforce; financing; information and research; and technology (19). The aim of the SELFIE framework is to support the development, implementation and evaluation of integrated care programs for multi-morbid populations (18). SELFIE uniquely focuses on aspects which are particularly important for multimorbidity, such as challenges of care fragmentation and needing care from multiple professionals across different sectors (18).

Our recent systematic literature review and meta-analysis, including 19 controlled intervention studies published between 2000 and April 2019, identified core components of nurse-led integrated care models for the home-dwelling older population; described patient, service and

process outcomes; and evaluated the impact of the care models (20). The majority of the studies did include the core components suggested in the SELFIE framework related to direct care delivery: the majority of the models were person-centered and proactive; included tailored holistic evaluations and informal caregiver involvement; shared decision-making and individualized care planning; and care was delivered by a multidisciplinary team, a dedicated core group of health professionals and a named lead coordinator. Risk screening to target the older people who would benefit most from an integrated care intervention was conducted in all but three studies, but all studies used different risk prediction tools. However, the study could not show convincing evidence regarding the beneficial impact on health and service outcomes (20), for example on long-term mortality, quality of life, functional status or hospitalisations. However, it must be noted that, first, a large heterogeneity was observed with respect to the included complex interventions. Second, heterogeneity was observed in term of outcomes and outcome measurements. Lastly, the majority of the studies included effectiveness outcomes only and lacked process and implementation outcomes hindering to determine whether the negative conclusions were due to intervention or implementation failure (20). These findings were consistent with similar systematic reviews for home-dwelling frail elderly, which have found major heterogeneity with respect to the target population, the study outcomes selected, the delivery of their intervention elements, and most importantly, the results found on a patient-, provider-, and system-level, impeding consistent conclusions (21-23). The lack of impact resulting from integrated care initiatives may be related to the outcomes measured and the measures used (21), but may also be a result of implementation issues with these complex interventions, potentially low fidelity to the intervention or the intervention lacking contextual fit (24). Therefore, this indicates the need for effectiveness studies which include process evaluations, contextual analysis, and measuring proximal implementation outcomes to determine if, how and why community-based integrated care for frail older adults is successful in practice (23).

Translation into real-world settings: Implementation Science

To facilitate the uptake of integrated care in daily practice (24) and overcome implementation issues, principles and methods from the field of **implementation science** should be incorporated into future research. Implementation science is “the scientific study of methods to promote the systematic uptake of research findings and other evidence-based practices into routine practice, and, hence, to improve the quality and effectiveness of health services” (25). Implementation science includes key elements to help bridge an intervention into the real-world (26), including for example, stakeholder involvement, conducting a contextual analysis, measuring implementation outcomes, the use of implementation strategies, and using hybrid implementation-effectiveness designs (27).

1.2 Canton Basel-Landschaft, a test field for Switzerland and Europe

Canton Basel-Landschaft (BL) has around 290,000 inhabitants and is currently the region with the second oldest population in Switzerland (28), with a predicted 43% and 79% increase of people aged 80 years or older by 2025 and 2040 respectively. In January 2018, the Volkswirtschafts- und Gesundheitsdirektion Basel-Landschaft published a new legal framework to redesign care for home-dwelling older people in the canton. This ‘Altersbetreuungs- und Pflegegesetz (SGS 941)’ mandates the reorganization of the 86 municipalities in Canton BL into larger care regions and the instalment of an ‘Informations- und Beratungsstelle (IBS)’ in each of these newly formed care regions. Currently, nine care regions have been informed including all 86 municipalities, named as follows: ABS, BPA Leimental, Rheintal, APG Laufental, Alter Birsstadt, Oberbaselbeit, Oberes Homburgertal, APG Liestal, Waldenburgertal plus. The legislation mandates the IBS to be staffed with at least a nurse who provides advice about ageing-related matters to the older population and assesses their care needs, when they or their family members visit the IBS, especially if and when an entry into a nursing home is planned. Subsequently, the INSPIRE research team has been working together with the Canton and the care regions to help operationalize and evaluate a care model for the IBS. The research team is **currently collaborating with the Leimental care region** (made up of 6 municipalities:

Bottmingen, Biel-Benken, Ettingen, Oberwil, Therwil and Burg im Leimental), to plan and implement the integrated care model in the IBS of this care region, known as the "**Fachstelle Betreuung Pflege Alter (BPA) Leimental**". The Fachstelle BPA Leimental is employing a Fachstelle manager, two nurses, a social worker, and administrative support.

INSPIRE: an implementation science project
The overall INSPIRE project is a **multi-phase implementation science project** which aims to develop, implement and evaluate an integrated care model for the IBS for home-dwelling older adults in Canton BL. The multi-phase study is designed in alignment with the recommendations of the Medical Research Council Framework for developing and evaluating complex interventions (17) (Figures 1, 2 and 3). INSPIRE incorporated many key elements from implementation science, including: iterative stakeholder involvement throughout the project duration; an intervention which is not only evidence-informed but also contextually-adapted; selecting and refining implementation strategies to support the intervention; and measuring both effectiveness and implementation outcomes (26).

The INSPIRE project has 3 phases:
Phase 1 (Figure 1): The **development** phase of the community-based **integrated care model** (i.e., **a complex intervention**) included three main activities: a literature review, a contextual analysis, and stakeholder involvement.

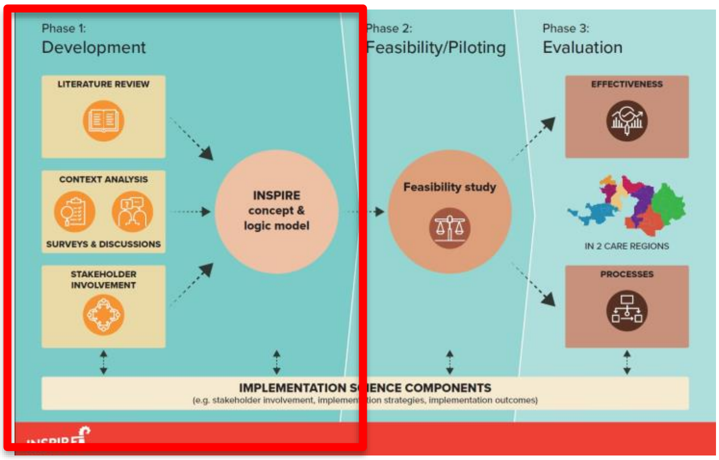

Figure 1: INSPIRE project - Phase 1 highlighted in red (adapted from Yip et al. [23])

As a result of these activities, we determined the **care model concept for the IBS/Fachstelle (Figure 2),** starting with the following **core component i) Screening** of older people for risk of frailty using a frailty screening tool, to identify the appropriate care they will require:

- Older adults with **low risk** of frailty will receive **health promotion and preventive care** from the Fachstelle nurse and/or social worker.
- Older adults **at risk** will receive **the remaining 3 core components of the intervention**, which are highlighted as part of an integrated care approach for frailty or multi-morbidity in the WHO's approach to *Integrated Care for Older People* (ICOPE) (29) and frameworks such as SELFIE (18), as well as our systematic review. These components include: **ii) a Comprehensive Geriatric Assessment (CGA)** <u>delivered by the Fachstelle nurse and social worker over the course of multiple appointments</u>, to identify the health and social care needs and goals of the older person; **iii)** Development of an **individualized care plan** by a multidisciplinary team, which will include evidence-based interventions and be coordinated by the Fachstelle nurse(s) and/or the social worker; and **iv) follow-up** depending on the situation of each older person, and adaptation of the individualized care plan, as needed.
- For those at very high risk and/or who have been sent to the Fachstelle with a recommendation from a health care professional for referral to a Nursing Home, the Fachstelle

Nurse together with the social worker will determine whether a Nursing Home referral is needed in close collaboration with the older adults' other professionals involved in their care.

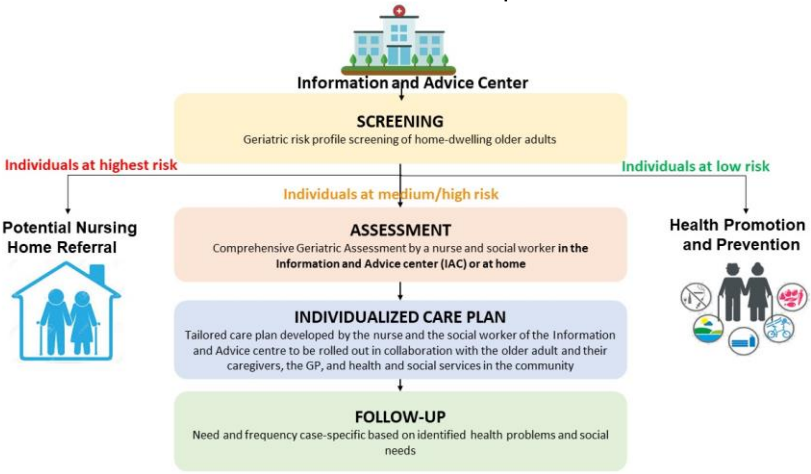

Figure 2. INSPIRE care model for the Fachstelle

Aspects of phase 1 were published (doi: 10.5334/ijic.5607) and previously submitted to the EKNZ under **EKNZ Project ID Req-2019-00900.**

Phase 2 (Figure 3): We will assess the feasibility of the community-based integrated model of care at the "**Fachstelle**" in **one care region**, **Leimental**. **The current ethics file refers to the feasibility study only (phase 2).**

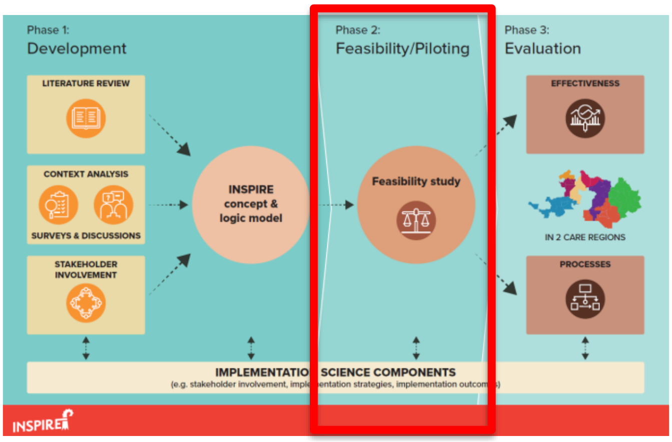

Figure 3: INSPIRE project - Phase 2 highlighted in red (adapted from Yip et al. [23])

Phase 3 (Figure 4): We will investigate the effectiveness of the care model with a pre-post study (work in progress, **not within the scope of this file**). A subsequent ethics file will be submitted for the effectiveness study (phase 3), pending adaptations based on the feasibility.

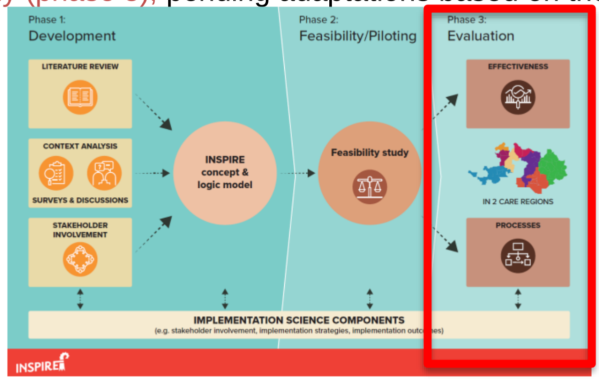

Figure 4: INSPIRE project - Phase 3 highlighted in red (adapted from Yip et al. [23])

Risk category
With regards to the risk categorization for the study, the study falls under the **category A**, meaning a low risk for the participants. The research study targets the feasibility of a care model at a new community-based center run by the care region of Leimental. According to HRO Art.7 (2) the planned measures for the researchers' collecting personal data entail only minimal risks and burdens, as it involves health and social care providers' participating in surveys; Fachstelle staff participating in meetings; older adults and informal caregivers' participating in interviews; personal and assessment data captured from the Fachstelle health records; as well as aggregated recruitment feasibility data. Measures are described below for any particularly vulnerable participants (e.g., frail older persons who may have varying levels of cognitive impairment).

2 PROJECT OBJECTIVES AND DESIGN

2.1 Hypothesis and primary objective

The primary objective of this feasibility study (Phase 2, Figure 3) is to **1) assess feasibility of recruitment to the Fachstelle and evaluate recruitment strategies 2) assess the adoption, acceptability, feasibility, and fidelity of the integrated care model at the Fachstelle,** and **3) explore perceptions** of older adults and their caregivers, Fachstelle staff, and health and social care providers (e.g., GP, Spitex Nurse) **towards the implemented care model,** and **if adaptations are needed** to the care model or the implementation strategies.

2.2 Primary and secondary endpoints

The current feasibility study (Phase 2 of INSPIRE project) **relies on multiple sample sets and data sources.**

Primary endpoints
<u>Implementation outcomes,</u> defined as "the effects of deliberate and purposive actions to implement new treatments, practices, and services" [31], will be captured from different sample sets, described in figure 5:

- **Adoption** is defined as the intention, initial decision, or action to try to employ a new intervention (31). Adoption will be qualitatively determined during the study through the **regular meetings** with the Fachstelle Nurse(s) and Social Worker ([sample 3]; see supplement **file 39E** for "Fachstelle staff meeting template"). These regular meetings will be led by the INSPIRE research team (e.g., Implementation Lead).
- **Acceptability** is defined as the perception among stakeholders that the intervention is agreeable (31). Acceptability will be captured qualitatively through **regular meetings** with the Fachstelle Nurse(s) and Social worker (sample 3) as well as through **interviews** with a nested sample of older adults (e.g., aged 75+, Groningen Frailty Indicator [GFI] score ≥4**,** received a CGA; see sample 1C) and their informal caregiver (sample 2; e.g., spouse, family member or neighbour), when possible. See supplement **file 39B** for the "interview guide-older adults" and supplement **file 39C** for the "interview guide-informal caregivers".
- **Feasibility** is defined as the perception among participating care providers that the intervention is feasible (31). Feasibility will be assessed for recruitment (e.g., referral to the Fachstelle) and aspects of the care model (i.e., screening, CGA, creation of a care plan and collaboration to coordinate care, and follow-up). Feasibility will be captured qualitatively in this study through a) the **regular meetings** with the Fachstelle staff (sample 3) and b) the **interviews** with a nested sample of participating older adults (sample 1C) and informal caregivers (sample 2), when possible. A final consensus meeting will be held at the end of the study with the INSPIRE research team, the Head of the Fachstelle and the Fachstelle Nurse(s) and Social Worker to confirm whether the care model is indeed "feasible" and we are ready to move into the effectiveness study.

- **Fidelity** is defined as the degree to which the intervention is implemented as it was designed in the original protocol (31). Fidelity will be quantitatively measured in this study phase by reviewing participating older adults' **Fachstelle health records** (see sample 1B**;** e.g., aged 64+, living at home, have a Fachstelle health record) to primarily determine if the care model components (i.e., screening, comprehensive geriatric assessment [CGA], care coordination and individualized care plan, and follow-up [see 1.2, Phase 1]) were delivered as intended. See **supplement file 39A** for the "fidelity tool" which will be used for the feasibility study. Fidelity will also be explored qualitatively in the regular meetings with the Fachstelle staff (sample 3).
- **Implementation processes related to collaboration between Fachstelle staff and external health and social care professionals** when coordinating care for an older adult, will be measured. The Normalization MeAsure Development **questionnaire (NoMAD**) (32) will be sent to external health and social care providers (e.g., GP, Spitex Nurse) who have collaborated with the Fachstelle staff for coordination of care of a participating older adult (sample 4, see 39D for **"**NoMAD Survey**"**). The NoMAD is a three-part (A, B, C), 23-item survey aimed at capturing implementation processes from the perspective of the professionals directly involved in the implementation of a complex intervention in healthcare (32). Part A (2-items) asks about their role and job category; in part B, two general questions about the intervention are rated with a response scale of 0-10 (0=not at all, 5=somewhat, 10=completely); and part C (19-items) is in the format of a 5-point Likert scale to indicate level of agreement (1=strongly agree, 3=neutral, 5=strongly disagree). There are four constructs assessed in Part C which are related to the Normalization Process Theory, being: *a) coherence* (4-items), *b) cognitive participation* (4-items), *c) collective action* (7-items; 1 of which we removed due to context), and *d) reflexive monitoring* (5-items). The NoMAD has a reliability of $\alpha = 0.89$ for the 20 items and has been cited in many research studies (32).

<u>Baseline factors</u>

<u>Individual characteristics</u> to describe the sample of consenting older adults (sample 1B) using the Fachstelle services (or having a home-based appointment) captured using the Fachstelle heath record:

Note: while a CGA includes multiple domains, the sections of the CGA which are described here are only the ones which relate to the description of the sample.

- **Demographic data:** age (year of birth), gender, educational level, and living situation (e.g., the number of people living with the older adult). This data will be extracted from the client's administrative forms.

- **Geriatric risk profile:** The Groningen Frailty Indicator (GFI) is a 15-item screening instrument aimed at determining the level of frailty (self-reported or interview-based) (33). It measures the loss of functions and resources in 4 domains: *a) physical* (mobility functions, multiple health problems, physical fatigue, vision, hearing), *b) cognitive* (cognitive dysfunction), *c) social* (emotional isolation), and *d) psychological* (depressed mood and feelings of anxiety) (33). All answer categories are dichotomized, where a score of 1 indicates a problem or dependency. The GFI score therefore ranges between 0 to 15, where a score of ≥4 represents moderate to severe frailty (33). This GFI screening tool will be administered by the Fachstelle staff routinely to individuals at the start of their first visit or at the home of the older adult. The reliability (internal consistency of 0.68) and validity (convergent: 0.45-0.61; discriminant: 0.08-0.50) of the GFI have also been reported (34). The GFI score will be extracted from the client's administrative forms.

- **Cognition:** As part of the CGA, the Fachstelle nurse will ask three screening questions to assess cognition, and determine whether further assessment is needed in the cognitive

domain: *1. Remember 3 words: flower, door, rice (for example) 2. Orientation in time and space: What is the full date today? Where are you now (home, clinic, etc)? 3. Recalls the 3 words?* These questions are part of a screening assessment for conditions associated with declines in intrinsic capacity, coming directly from the World Health Organization handbook: Integrated care for older people (ICOPE): guidance for person-centred assessment and pathways in primary care (35). If further assessment is needed, the Mini-Cog will be performed. The Mini-Cog is an instrument which takes 3-minutes to complete, and helps to detect whether an older adult has cognitive impairment. There are two components included: a 3-item recall test for memory and a clock drawing test. The creators recommend a cut score for dementia screening of 0-2 = positive; 3-5 = negative. However, when looking for greater sensitivity, a cut point of <4 is recommended as it may indicate that further evaluation of cognitive status is needed (36). Tsoi et al reported the pooled sensitivity and specificity of the Mini-Cog as 0.91 and 0.86, respectively, and highlighted that it is relatively simpler and shorter than the MMSE, which is the most widely applied clinical test for dementia screening (37). Data available on cognition will therefore be extracted from the Fachstelle health record, if a CGA was performed.

- **Depressive symptoms:** As part of the CGA, the nurse will ask the two screening questions, as recommended by the ICOPE, to assess depression (i.e., "*1. over the past two weeks, have you been bothered by... feeling down, depressed or hopeless; 2. ...little interest or pleasure in doing things?"*), and determine whether further assessment is needed (35). As recommended by the ICOPE (35), if further assessment of depressive symptoms is needed, an assessment of mood is conducted which includes asking about problems related with: sleep, energy, eating, feeling bad/failure, concentration, slow movements, restlessness, and harmful thoughts. These questions assessing mood come from the Patient Health Questionnaire (PHQ-9). As described in the ICOPE (35), "if a person has at least one of core symptoms and one or two additional symptoms, they may have depressive symptoms. If a person has more than two symptoms, they may qualify for a diagnosis of depressive disorder". Data available on presence of depressive symptoms will therefore be extracted from the Fachstelle health record, if a CGA was performed.

- **Multimorbidity:** During the CGA, the nurse will ask older adults about some of their major health concerns or diseases for which they take medication. The nurse will also ask for permission to follow-up with the older adults' GP to confirm any chronic illnesses/conditions the person has. The data available on presence of multi-morbidities will therefore be extracted from the Fachstelle health record, if a CGA was performed.

- **Nutritional status:** During the CGA, the Nurse will ask two screening questions to assess malnutrition ("*1. Have you unintentionally lost more than 3 kg over the last three months? 2. Have you experienced loss of appetite?"*), and whether further assessment is needed, as recommended by the ICOPE (35). If further assessment is needed, the Mini Nutritional Assessment – short form (MNA-SF) will be performed by the Nurse, which is a validated nutrition screening and assessment tool that can identify older people who are malnourished or at risk of malnutrition (38). It contains 6 screening questions and the sum scores range between 0 and 14. Individuals scoring between 12 and 14 are considered to have a normal nutritional status, whereas those scoring between 8 and 11 are considered at risk of malnutrition, and between 0 and 7 are considered malnourished. The sensitivity (94.0%) and specificity (83.3%) of the MNA-SF have been reported by Lilimand et al, when validating the MNA-SF and comparing to the full MNA (39). The data available on nutritional status will therefore be extracted from the Fachstelle health record, if a CGA was performed.

Fall history: A fall incident will be defined as "an unexpected event in which the patient comes to rest on the ground, floor or lower level" (World Health Organization, 2018). It will be assessed by asking if they had two or more falls in the previous 12-month period (40). The data available on fall history will be extracted from the Fachstelle health record, if a CGA was performed.

Secondary endpoints

Recruitment feasibility and recruitment strategy measures, using the framework by Stewart et al. (30), will be provided by the Fachstelle administrative staff:

External processes: monitoring **outreach strategies** used to promote the Fachstelle to older adults (e.g., letters, brochures) and to sources who could refer/recommend the Fachstelle to older adults (e.g., in-person meetings with Hospitals or Spitex) as well as respondents to the outreach strategies (e.g., # of Hospitals who administer flyers to their staff).

Internal processes: summarized information related to **Fachstelle use** of all visitors/home appointments (sample 1A **-** *see Table 2 for criteria)*, including:

- **number of visitors/home appointment users**
- **sociodemographic data of all visitors/home appointments users:** age, gender, municipality of residence
- **reason for their appointment/contacting the Fachstelle** and **referral source** (i.e., how they heard about the Fachstelle, and/or the organization which referred them)
- **type of service received by visitors/Fachstelle users**: a) health promotion and prevention; b) a full CGA; c) a brief assessment to confirm whether a nursing home referral is warranted; or d) other

Note: "Visitors/users" describes those either attending the Fachstelle in person or contacting the Fachstelle to receive the services at home

The **endpoints are described in Figure 5 and in Table 1 below**. **Figure 5 describes the flow of selected sample sets** for the collection of recruitment-related data, individual characteristics of consenting older adults who use the Fachstelle services, and the implementation outcomes as follows:

•

**Variables**	***Sample set (label of sample set)**	**Data source**	**Tools**
Recruitment feasibility and strategies - External processes			
Outreach strategies to promote the Fachstelle (to GPs, Hospitals, older adults, etc.)	N/A	• Fachstelle administrative data	N/A
Respondents to outreach strategies			
Recruitment feasibility and strategies- Internal processes			
# of visitors[a] and appointments	• All visitors[a] to the Fachstelle (1A)<br><br>*Note: Visitors[a] = either attending the*	• Fachstelle administrative data	N/A
Visitors'[a] socio-demographics (age, gender, municipality of residence)			

Reason for appointment/contacting the Fachstelle & Referral source	*Fachstelle in person or contacting the Fachstelle to receive the services at home*		
Type of service received by visitors[a]			
Implementation outcomes			
Adoption	• Fachstelle staff (3)	• Fachstelle staff meeting log	N/A
Acceptability	• Fachstelle staff (3)<br>• Older adults (1C)<br>• Informal caregivers (2)	• Fachstelle staff meeting log<br>• Older adults & informal caregiver interviews	N/A
Feasibility	• Fachstelle staff (3)<br>• Older adults (1C)<br>• Informal caregivers (2)	• Fachstelle staff meeting log<br>• Older adults & informal caregiver interviews	N/A
Fidelity	• Older adults (1B)<br>• Fachstelle staff (3)	• Fachstelle health record<br>• Fachstelle staff meeting log	N/A
Implementation processes	• External collaborators (4); described in Table 2	• Questionnaire	• NoMAD (32)
Individual characteristics of consenting older adults (1B)			
Demographic data	• Older adults (1B)	• Fachstelle health record	N/A
Geriatric risk profile			• Groningen Frailty Indicator (33)
Cognition			• ICOPE Screening Questions (35) and Mini-Cog (36)
Depressive symptoms			• ICOPE Screening Questions and mood assessment (35)
Multimorbidity			N/A
Nutritional status			• ICOPE Screening questions and Mini Nutritional Assessment – Short Form (39)
Fall history			• Fall screening

Table 1. Summary of study variables and data sources

**Figure 5 illustrates the flow of sample sets listed in table 1*

2.3 Project design

The feasibility study (Phase 2 of INSPIRE project) will be conducted in March 2022 (estimated to be within a few weeks of the official opening of the Fachstelle), using multiple methods. For objective 1, a descriptive study will be conducted to monitor the strategies used to promote the Fachstelle and to assess which ones were effective. To address objectives 2 and 3, which are the core aspects of this study phase, a parallel convergent mixed methods observational design will be used (whereby both quantitative and qualitative data are collected during a similar time period, analyzed separately, then merged). The quantitative data sources will include: Fachstelle administrative data, Fachstelle health records, and the NoMAD survey, while qualitative sources will include: interviews and meeting logs. The study will take place in a single center (i.e., the Fachstelle in the Leimental care region). Effectiveness outcomes are not within the scope of this study phase.

3 PROJECT POPULATION AND STUDY PROCEDURES

3.1 Project population, inclusion and exclusion criteria

Figure 5 and Table 1 (above) described the multiple sample sets and data sources used in this study. Further details are described here in Table 2.

Sample label	Sample set*	Description of sample	Inclusion/exclusion criteria	Type of data assessed through each sample
1A	All individuals visiting/contacting the Fachstelle (center or home)	All individuals (e.g., older adults and their family member) who visit the Fachstelle or have a home-based appointment		• Recruitment feasibility data
1B	Older adults	A consecutive sample of older adults who are eligible for review of their Fachstelle health record	*Inclusion criteria:*<br>• Aged 64 years or older (*as this is the statutory retirement age for women in Switzerland and the minimum age of the group we expect to use the Fachstelle services*)<br>• Living at home<br>• German or English-speaking<br>• Providing Individual/Proxy Informed Consent (See "3A Informed Consent – Older adult"/"3B Informed Consent -Relative").<br>• Had a Fachstelle health record created<br><br>*Exclusion criteria*:<br>• Residing in a nursing home or planned permanent admission to a nursing home<br>• Receiving end-of-life care	• Individual characteristics<br>• Implementation outcomes (fidelity)

1C	Older adults	Using a purposeful sampling strategy, a nested sample of older adults will be interviewed	*Inclusion criteria*:<br>• Aged 75 years or older<br>• Living at home<br>• Living in the participating care region Leimental<br>• English or German-speaking<br>• **Groningen Frailty Indicator [GFI] ≥4**<br>• If the older adult had a CGA by the Fachstelle staff<br>• Providing Individual/Proxy Informed Consent (See **3A** “Informed Consent – Older adult”/ **3B** ”Informed Consent -Relative”)<br><br>*Exclusion criteria*:<br>• Residing in a nursing home or planned permanent admission to a nursing home<br>• Receiving end-of-life care<br>• Participation in another study with health-related interventions within the 30 days preceding or during the present study<br>• GFI < 4<br>• If the older adult did not have a CGA by the Fachstelle staff	• *Individual characteristics will have already been collected in 1B*<br>• Implementation outcomes (acceptability, feasibility)
2	Informal caregivers	Informal caregivers of older adults who participate in an interview	*Inclusion criteria:*<br>• Individuals who attended a Fachstelle appointment (in the center or at home) with a participating older adult.<br>• The older adult must agree for the INSPIRE research team to contact the informal caregiver.<br><br>*Exclusion criteria:*<br>• Individuals who did not attend a Fachstelle appointment with a participating older adult or who were not present in a home visit by Fachstelle staff (as the interview questions focus on their perception of the Fachstelle’s feasibility and acceptability)<br>• Any individuals whom the older adult did not agree for the INSPIRE research team to be contacted	• Implementation outcomes (acceptability, feasibility)

3	Fachstelle nurse(s) and social worker	Who work at the Fachstelle to provide services for older adults	*Inclusion criteria:*<br>• Individuals employed by the Fachstelle BPA Leimental as a Nurse or Social Worker<br><br>*Exclusion criteria:*<br>• Other individuals employed by the Fachstelle BPA Leimental (e.g., Administration)	• Demographic data<br>• Implementation outcomes (adoption, acceptability, feasibility, fidelity)
4	External collaborators	External collaborators (health and social care collaborators e.g., GP, Spitex Nurse)	*Inclusion criteria:*<br>• Individual health or social care providers who are indicated in the Fachstelle health record as having worked together with the Fachstelle nurse(s) or social worker in the care coordination of a participating older adult<br><br>*Exclusion criteria:*<br>• Individual health or social care providers who have not contributed to the coordination of care with the Fachstelle staff for a participating older adult	• Implementation outcomes (implementation processes directly related to collaboration between Fachstelle staff and external health and social care professionals)

Table 2. Description of sample sets and inclusion/exclusion criteria

*Note for Sample 1B + 1C: Inclusion or exclusion does not impact whether the individual still receives the standard of care in the Fachstelle, as this is guaranteed regardless of study participation.

3.2 Recruitment, screening and informed consent procedure

3.2.1 Screening and recruitment

Objective 1 (Recruitment feasibility and strategies): external processes, including Fachstelle outreach, are not conducted by the researchers. For internal processes, all *Fachstelle visitors (*sample 1A*)* will be included in our sample. Recruitment-related data will by aggregated and sent to the INSPIRE research team by the Fachstelle administrative staff, hence no recruitment/screening is needed.

Objectives 2 & 3*: Older adults* [sample 1B & 1C] *and relatives/legal representatives*
Recruitment will be done following these steps:
(i) **Older adults' arriving at the Fachstelle/receiving an appointment** at their home, will be asked to fill in client information forms as part of his/her Fachstelle health record, including the GFI screening tool (self-administered). The GFI score will help the nurse(s) to determine whether the older adult needs:
a) GFI < 4 = health promotion and prevention (no CGA); or
b) GFI ≥ 4 = full CGA
The GFI score will therefore also determine whether the INSPIRE research team will invite the older adult for an interview.

(ii) For home-based older adults aged 64+ who have a health record created (sample 1B), the Fachstelle nurse will inform them about the study and provide the INSPIRE study invitation letter (offering to read the letter for older adults depending on their preference; see "**11A- INSPIRE Recruitment letter – older adults**"). **The nurse will ask if they are interested and agree for their contact information to be passed to the INSPIRE team**. On a weekly basis, the INSPIRE team will reach out to the Nurse to identify potential participants for a Fachstelle health record review (based on inclusion/exclusion criteria).

(iii) **The research team representative will then reach out to confirm the invitation to the older adult and ask for consent for participation**. The INSPIRE research team will either approach the older adult in the Fachstelle, their home or by phone to explain the study procedures and ask for consent to review their Fachstelle health record and to potentially participate in an interview (see "**39F- script – health record review**" and "**3A- informed consent older adults"**). Consent will be documented by signature. After review of the Fachstelle health record, only eligible older adults (e.g., aged 75+, CGA completed, GFI≥4; Sample 1C) will be contacted again to arrange an interview.

Proxy consent, if required: The nurse will assess cognition as part of the regular standard of care during the CGA. If the nurse is concerned about the older adults' capacity to consent to the research study based on the Mini-Cog assessment and their clinical judgement, a proxy consent will be sought (i.e., legal representative). In this case, when the nurse introduces the study to the older adult, he/she will also ask if the INSPIRE research team can reach out to their legal representative as well.
The INSPIRE research team will speak to the family member/legal representative and older adult, and will ask for proxy consent (see "**3B- Informed consent, relative"**). If a proxy has consented for the interview, the interview can also be in presence of the proxy. If the proxy provides consent for the health record review but not for the interview (or if the individual does not want to complete an interview), the proxy will still be invited to complete the interview designed for informal caregivers.

Informal caregiver [sample 2]
If an informal caregiver (e.g., family member or neighbour) attended the Fachstelle appointment with an older adult, the older adult will be asked if we could also interview the informal caregiver to gather their perspective on the older adults' experience with the Fachstelle. Given the crucial role informal caregivers play in the care and support of frail older adults who are living at home, it is beneficial to gather the informal caregivers' perspective of the feasibility and acceptability of the Fachstelle appointments and if there were any barriers to participation. If the older adult agrees to an informal caregiver being contacted, the INSPIRE team will contact the informal caregiver to discuss the study procedures, the consent form (**see "3C – Informed consent - informal caregiver**"), and arrange a time for the interview (see "**39C – Interviews with informal caregivers**"). The consent of the informal caregiver may be by signature, depending on the caregivers' preferences for the interview location.

Fachstelle nurse(s) and social worker [sample 3]
The INSPIRE research team (i.e., Implementation Lead) will give the Fachstelle staff an invitation letter see ("**11B- INSPIRE Recruitment letter - generic**") and a consent form (see "**3D-Informed consent Fachstelle staff"**), inviting them to participate in regular meetings with the research team during the feasibility study. The purpose of these meetings will be to regularly explore their perceptions of the Fachstelle with regards to adoption and feasibility of the care model, fidelity, and additional implementation strategies needed.

External collaborators [sample 4]
As part of the marketing of the Fachstelle, the Fachstelle manager intends to contact external collaborators (i.e., existing community health and social providers who may have collaborated with the Fachstelle staff for care coordination of a visiting older adult, such as GPs or Spitex

Nurses) together with the INSPIRE team, reminding them that the Fachstelle and INSPIRE are working together, and that the INSPIRE team may reach out to them separately. The INSPIRE team will then extract the contact information of collaborating external professionals from the Fachstelle health records of participating older adults. **INSPIRE will contact the collaborating professionals and send them a study invitation letter** (see "**11B- INSPIRE Recruitment letter**", which specifies that completion of the survey implies their consent) and the NoMAD survey by email (or by paper as an alternative option). The NoMAD aims to assess the Fachstelle implementation processes regarding the collaboration for care coordination. It does not collect any identifying information and only summarized results will be reported.

No compensation will be given to any study participants.

3.3 Study procedures

Three to four weeks after the opening of the Fachstelle, which is estimated to open in January/February 2022, the feasibility study will start.

As there are multiple sample sets in this study, **Figure 6** describes the data collection process from samples 1A, 1B and 1C.

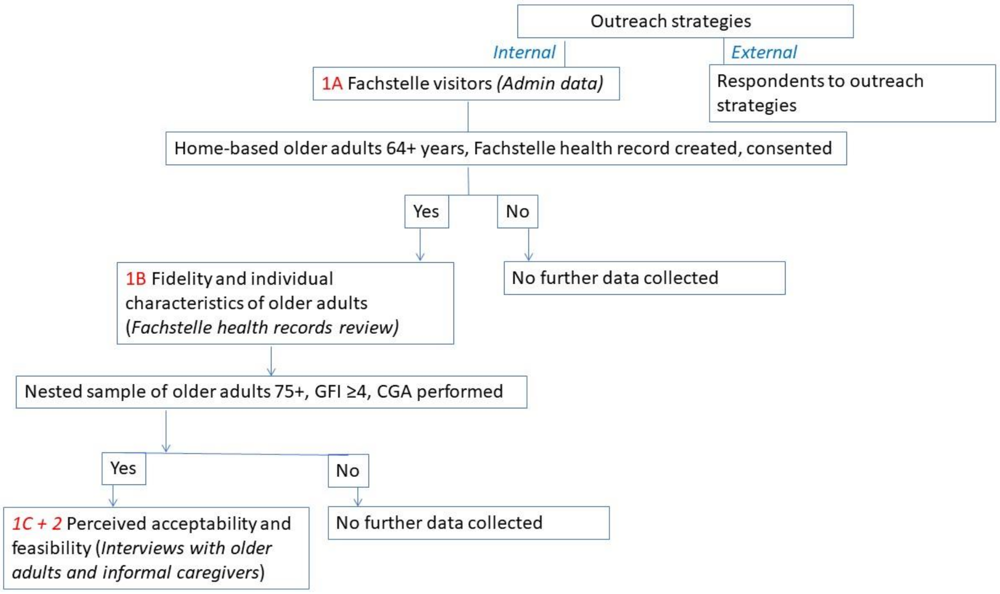

Figure 6. Flow chart for data collection from samples 1A, 1B + 1C

1. **Recruitment feasibility and strategies- *external processes***: INSPIRE will monitor all Fachstelle promotion activities (e.g., # of letters sent, # of meetings with external groups) and respondents (e.g., # of Hospitals who administer flyers to their staff) at the end of each month during the feasibility study, which will be provided by the Fachstelle.

Recruitment feasibility and strategies- *internal processes*: In March 2022, when the feasibility study starts, the Fachstelle administration will provide the INSPIRE research team with summarized administrative data (demographic and Fachstelle use data about all visitors or home appointments) (sample 1A). As part of regular practice, when an older adult arrives at the Fachstelle (or has an appointment at home), they will be asked to complete the standard client information forms and a Fachstelle health record will be created.

2. If the individual is eligible (e.g., **home-based older adult aged 64+ with a Fachstelle health record**; see criteria for Sample 1B), the Fachstelle nurse will invite them to participate in the INSPIRE Feasibility Study. The Fachstelle nurse will provide the original invitation to the study, while the INSPIRE research team will explain the study, the consent form and conduct the data

collection. Proxy consent will be asked for if indicated by the Fachstelle Nurse. Participation in this portion of the feasibility study entails completing an informed consent form and allowing the INSPIRE research team to extract data from their Fachstelle health record to assess fidelity (based on the assessments conducted) and capture variables which describe the sample. The INSPIRE team will review the Fachstelle health records of participating older adults (sample 1B) each week and once at the end of the study to complete the fidelity tool.

3. The informed consent form (described above) also specifies that the individual may be invited to participate in an interview, as the research team will interview a nested sample (sample **1C**) of 8-12 older adults. If the older adult is eligible for an interview based on review of their Fachstelle health record (e.g., **aged 75+, had a GFI ≥4 and had a CGA**), the INSPIRE team will follow-up with the older adult to invite them for the interview. The interview will be approximately 45 minutes and will take place in the Fachstelle or the older adult's home within two-weeks after their second appointment, to gather their perceptions of the care model.

4. At the end of the interview, if an informal caregiver attended the appointment with the older adult, the older adult will be asked if we could invite the informal caregiver for an interview as well (to understand if the informal caregiver perceives the Fachstelle care model to be acceptable and feasible, and if they identify any barriers/facilitators for the older adults' participation in the care model). The INSPIRE team will then reach out to the informal caregiver to provide the study information and consent.

If a proxy has provided consent for the health record review but not for the interview (or if the older adult does not want to complete an interview), the proxy will still be invited to complete the interview designed for informal caregivers.

5. External providers who are identified in the Fachstelle health records and collaborate with the Fachstelle will also be asked to complete one questionnaire, the NoMAD, at the end of the feasibility study, via an email link to the survey or alternatively in-paper (according to preferences).

6. Throughout the feasibility study, regular meetings will be held with the Fachstelle staff, and one consensus meeting will be held with Fachstelle staff at the end of the feasibility study.

7. All data related to the feasibility study will be collected within 2-3 months. Therefore, data collection will end at the latest by May 31st, 2022.

Data collection or extraction:

1) Recruitment feasibility data

- External processes data: At the end of each month during the study, INSPIRE will monitor all Fachstelle promotion activities and respondents (provided by the Fachstelle)
- Internal processes data: The INSPIRE team will capture data provided by the Fachstelle administration at the end of each week, or a mutually agreed time
- Data will be saved on the research drive of the Institute of Nursing Science

2) Fachstelle health records (of study participants only)

- A member from the research team will visit the Fachstelle weekly to extract sample characteristics and fidelity data **("39A – Fidelity tool"**), using Case Report Forms programmed in Castor, a secure online server (see 7.1)
- At the end of the study, Fachstelle health records will be accessed again to check if follow-up occurred within the timeframe indicated

3) Interview notes and audio recordings with older adults (and informal caregiver, if possible)

- Conducted within two-weeks of their second appointment

- Interview notes will be hand-written and scanned into Castor
- Audio recordings and paper-based notes will be saved in a locked cabinet in the Institute of Nursing Science

4) Meeting logs and audio recordings of meetings with Fachstelle Nurse(s) and social worker

- Regular meetings will be held
- Meeting logs will be hand-written and scanned into Castor
- Audio recordings and paper-based notes will be saved in a locked cabinet in the Institute of Nursing Science

5) NoMAD questionnaire for collaborating health and social care providers

- Administered towards the end of the study via email through a LimeSurvey link
- Results will be stored in LimeSurvey, downloaded and saved on the research drive of the Institute of Nursing Science
- If paper-based survey requested, sent via paper and results will be directly entered into LimeSurvey by INSPIRE research team. Paper-based copy will be stored in a locked cabinet of the Institute of Nursing Science.

3.4 Withdrawal and discontinuation

Participation in the study is voluntary. Participants (or the proxy) may withdraw at any time throughout the study period by contacting the INSPIRE project team (contact information provided in the study invitation letter and consent form). Participants who withdraw for research follow-up after having consented will be informed that the data collected until that point will be saved and stored in pseudonymized form. After their data is entered into Castor, their individual data will be anonymized, as their key assignment linking the research data with their identity will be destroyed. Hence, the already collected data will be analyzed but they will no longer be contacted for research follow-up.

4 STATISTICS AND METHODOLOGY

4.1. Statistical analysis plan

Sample size

Recruitment of samples 1A to 4 for the feasibility study will last for approximately two to three months (starting 3-4 weeks after the Fachstelle opens in Leimental).

a) **All visitors** to the Fachstelle will be included in the assessment of Fachstelle use (1A)
b) **All eligible older adults** who have a Fachstelle health record created during this time will be invited to participate in the study for review of their Fachstelle health record (1B). While it is currently not possible to provide a precise estimate of the number of visitors to this new center, we estimated a maximum of 18 older adults, estimating 1-2 older adults who visit the Fachstelle each week over a period of 2-2.5 months that will be willing to participate in the study.
c) As we will interview a nested sample (1C), we estimated the sample size for the interviews to be approximately 8-12 older adults. Because analysis will be occurring in parallel with data collection, we estimate information power could likely be achieved in a minimum of 8 interviews (41).
d) We estimate approximately 8-12 informal caregivers (2) [1 per older adult participating in an interview].
e) We estimate 3 Fachstelle staff (3) (i.e., 2 Fachstelle Nurses and 1 Social Worker) to participate in the study.
f) We estimate 18 external collaborators (4), who were involved in the care of the

aforementioned 18 older adults, will participate in the study.

Quantitative analysis of feasibility study

Descriptive statistics (e.g., mean, median and SD) and frequencies will be calculated and reported to describe the data on recruitment feasibility, participant demographics, study fidelity and survey results.

Qualitative analysis of interviews in the feasibility study

Qualitative data from the interviews with participating older adults (as well as their informal caregivers, if possible) will be analyzed using rapid qualitative analysis, as described by Hamilton (42). Two INSPIRE researchers will participate in the interview and take notes on a pre-structured meeting log consistent with the conceptual model domains. A template will be prepared *a priori* with the main topics to summarize the researchers' meeting notes (42). These notes will be coded, analyzed for themes, and further analyzed using a matrix analysis (42). Interviews will also be audio recorded should further verification or transcription be needed. The main actionable findings will be shared to guide further implementation in real time.

Qualitative analysis of meeting logs in the feasibility study

Qualitative data from the meeting logs with the Fachstelle nurse(s) and social worker will be analyzed using rapid qualitative analysis (42), following a similar procedure as described above.

Integration of quantitative and qualitative data

Quantitative and qualitative data will be collected and analyzed in parallel before the quantitative and qualitative results will be merged through side-by-side comparisons in joint displays or in discussions.

4.2. Handling of missing data

Missing data will be documented and patterns analyzed.

5 REGULATORY ASPECTS AND SAFETY

5.1 Local regulations / Declaration of Helsinki

This research project will be conducted in accordance with the protocol, the Declaration of Helsinki (43), the principles of Good Clinical Practice, the Human Research Act (HRA) (1) and the Human Research Ordinance (HRO) (2) as well as other locally relevant regulations. The Project Leader acknowledges his responsibilities as both the Project Leader and the Sponsor.

5.2 Notification of safety and protective measures (HRO Art. 20)

The project leader is promptly notified (within 24 hours) if immediate safety and protective measures have to be taken during the conduct of the research project. The Ethics Committee will be notified via BASEC of these measures and of the circumstances necessitating them within 7 days.

5.3 Serious events (HRO Art. 21)

If a serious event occurs, the research project will be interrupted and the Ethics Committee notified on the circumstances via BASEC within 7 days according to HRO Art. 21[1].

5.4 Procedure for investigations involving radiation sources

Not applicable

5.5 Amendments

Substantial changes to the project set-up, the protocol and relevant project documents will be submitted to the Ethics Committee for approval according to HRO Art. 18 before implementation. Exceptions are measures that have to be taken immediately in order to protect the participants.

5.6 End of project

Upon project completion or discontinuation, the Ethics Committee is notified within 90 days. The participant Master file will be locked in a cabinet at the Institute of Nursing Science for a duration of 10 years and destroyed thereafter. Any paper-based notes and surveys (if applicable) will be treated confidentially, and kept locked in a cabinet at the Institute of Nursing Science for a period of 10 years and destroyed thereafter. Electronic files which are securely saved on the Institute of Nursing Science research drive will be stored for a duration of 10 years and destroyed thereafter. The audio files will be destroyed at the end of the study. The data entered in Castor will be stored for at least 25 years (depending on local laws).

5.7 Insurance

In the event of project-related damage or injuries, the liability of the University of Basel provides compensation, except for claims that arise from misconduct or gross negligence.

6 FURTHER ASPECTS

6.1 Overall ethical considerations

Participation in the 'Feasibility of a community-based integrated care model for older adults living at home' study will be of voluntary nature.

The invitation letter for the Feasibility Study will inform older adults about the study requirements (i.e., allow the research team to access the Fachstelle health record, and potentially complete an interview) and that their participation is voluntary. The consent form indicates that their decision to participate or not does not have any negative consequences about their access to care or services. Informed consent will be given to the interested participants for asking their consent to use their data for the primary objectives of the project. We will explain that the data collected is not of the nature that we will be able to provide medical advice or make diagnoses requiring us to inform the participant.

While a CGA can typically be time-intensive for an older adult when conducted in a hospital-based setting, we have revised our approach for the community-setting, where the CGA will be split into 2 appointments. We anticipate this CGA process to also be shorter overall for some older adults, as demonstrated by our initial testing. The amount of time it will take for the CGA will continue to be monitored by the INSPIRE research team and will be re-evaluated.

---

[1] A serious event is defined as any adverse event where it cannot be excluded, that the event is attributable to the sampling of biological material or the collection of health-related personal data, and which:
a. requires inpatient treatment not envisaged in the protocol or extends a current hospital stay;
b. results in permanent or significant incapacity or disability; or
c. is life-threatening or results in death.

The invitation letter and consent form for the Fachstelle Nurse(s) and Social Worker and the external care providers will indicate that participation is voluntary, and that there will be no negative consequences if they choose not to participate.

The following documents will be pilot tested by each respective target population to ensure comprehension, flow and relevance: the study recruitment letter, the interview guide for older adults, the interview guide for informal caregivers, and the NoMAD survey.

Participation in the project is considered of low risk as it involves permission to review a Fachstelle health record, completion of surveys, participation in interviews/meetings and analysis of administrative data. All participant data collected will be handled confidentially and will be stored securely, using a code. Only authorized members of the research team will have access to the master file which includes any participant's personal information.

6.2 Risk-Benefit Assessment

Participation in the 'INSPIRE Feasibility Study' is considered to be of minimal risk for the individuals. Risks for older adults include feeling unpleasant thoughts or emotions from recalling past experiences and reflecting on future care needs. Participants will not receive any individual benefits, given that all individuals at the Fachstelle will receive the same intervention (which we anticipate will be of benefit to them). However, participants could experience a positive feeling of contributing to society, as we will explain to them that by granting their participation, it will enhance our potential to successfully implement and evaluate integrated care in the participating care regions of Canton BL.

6.3 Rationale for the inclusion of vulnerable participants

Although a vulnerable group, older persons are the target of this project for this reason. Because the older population is growing and care is becoming more complex, stakeholders need to adapt the healthcare and social system to responds to the needs of this population. The care model designed by the INSPIRE research team aims to be inclusive of all older people, helping to determine their needs and coordinate their care so that they can continue to live at home with appropriate support.

We anticipate that some older adults who visit the Fachstelle/have an appointment at home may have varying levels of cognitive impairment. A-priori excluding the group of older adults who have cognitive impairment would significantly impact the validity and generalizability of the study given that this is an increasing group of home-dwelling older people as a result of demographic ageing and rising prevalence of dementia. Procedures will be followed to request proxy consent for these individuals.

An invitation letter will be provided together with the consent form, which participants will be asked to review before deciding whether or not to participate in the project. The information sheet includes the contact information of INSPIRE where participants can reach us if they have questions or concerns about the project.

7 QUALITY CONTROL AND DATA PROTECTION

7.1 Quality measures

Study Members:

- All members of the INSPIRE research group (i.e., research team from the Institute of Nursing Science at the University of Basel which includes a PI, Academic Lead, Implementation Lead, 3 PhD students and Research Assistants/Master's students) who participate in the data collection process will be trained on study-specific procedures.

Data Storage:

- There will be five elements of data collection in this study: 1) Feasibility of recruitment (external and internal processes data), 2) Fachstelle health records of participating older adults and fidelity tracking of assessments completed in the health records; 3) interviews with participating older adults and their informal caregivers (if possible); 4) regular meeting logs with Fachstelle staff and 5) questionnaires of external care professionals.
- Some study data (i.e., fidelity data and information from the Fachstelle health record; demographic information; the researchers' scanned interview notes; the researchers' scanned meeting logs with Fachstelle staff) will be entered/uploaded into CASTOR EDC (Electronic Data Capture), which is a secure online server; it is protected from unauthorized access and access to the database is only possible with personal access data. This data will be double checked by a second individual from the research team (i.e., a PhD student or a Research Assistant/Master's student). Backups of the servers take place twice a day and are stored in different geographical locations to ensure maximum security and continuity. Regarding the legal regulations to be fulfilled, the following paragraph can be quoted: "Castor complies with all applicable laws and regulations, including ICH E6 Good Clinical Practice (GCP), 21 CFR Part 11, EU Annex 11, General Data Protection Regulation (GDPR), HIPAA (US), ISO 9001 and ISO 27001. By using Castor, researchers are enabled to comply with these laws and regulations. Castor is a validated system, and approved by external auditors" (Castor EDC, 2020). The database fulfils all requirements of the HFG.
- Other electronic data files (i.e., Fachstelle external and internal processes data in Excel; scanned PDFs of the researchers' interview notes; the Fachstelle staff meeting logs), and the generated excel sheets containing survey results from LimeSurvey (note: LimeSurvey server located in Germany, where full EU GDPR compliance is guaranteed) will be stored on the secure server of the Institute of Nursing Science, where 4 hourly back-ups occur and are mirrored in two different locations and access is controlled by the Active Directory of the University of Basel, which prevents misuse.
- The recordings of the interviews with older adults and informal caregivers, and the audio recordings of the meetings with Fachstelle staff will be securely stored in a locked cabinet at the Institute of Nursing Science, which only two members of the research team will have access (i.e., the Implementation Lead and Academic Lead). The recordings will be saved for a period of 10 years and destroyed thereafter. The paper-based files of the interview notes, the Fachstelle staff meeting notes, and any of the NoMAD questionnaire will also be stored in a locked cabinet at the Institute of Nursing Science.
- For quality assurance, the Ethics Committee may visit the research sites. Direct access to the source data and all study related files is granted on such occasions provided that all involved parties keep the participant data strictly confidential.

7.2 Data recording and source data

Data sources:

1) Recruitment feasibility data:

> a) Fachstelle external processes data will be provided by the Fachstelle administration and entered in an Excel file by the INSPIRE research team
>
> b) Fachstelle internal processes (i.e., Fachstelle use data and demographics): the Fachstelle administrative staff will provide this administrative data to the INSPIRE research team who will enter it into an Excel file

2) Fachstelle health records: the research team will use an electronic case report form (eCRF) for each enrolled older adult in Castor. The older adult will be assigned a unique code (e.g., location – gender – number; "LEIMM1"), to ensure the data is pseudonymized. The first section of the eCRF will include the relevant health-related information to report participant characteristics. The second section of the eCRF will include the fidelity data, based on a review of the Fachstelle health record to document the completed assessments and processes. The

eCRF will be filled in by 1 or 2 INSPIRE research team member(s) (i.e., the Implementation Lead or a Research Assistant).

3) Older adult interviews: the researchers' interview notes will be written by hand, scanned and uploaded as an attachment to the eCRF. There will be no identifiable information on the interview notes and only the unique code will be used. If an older adults' informal caregiver also participates in the interview, the written notes will be uploaded as an attachment in the eCRF. The interviews will also be audio recorded.

4) Meeting logs with Fachstelle staff: One eCRF will be completed for each meeting. The researchers' notes obtained through the meetings with Fachstelle staff will be written by hand, scanned and uploaded as an attachment. Demographic data of the Fachstelle staff, which are collected once, (i.e., years of experience and education) will be typed into the eCRF.

5) Questionnaires of external care professionals: The NoMAD questionnaire will be administered via e-mail using a LimeSurvey link (or alternatively available via paper, if requested), and the results stored within LimeSurvey (which have no identifiable information). If any copies are completed by paper, a PhD student or Master's student/research assistant from INSPIRE will enter the survey results directly into LimeSurvey.

For data and query management, monitoring, and reporting, CASTOR EDC will be utilized. This internet-based secure database is developed in agreement with the Good Clinical Practice (GCP) guidelines. The researchers will assure that all data in the course of the study will be entered completely and correctly in this respective database. Access, editing and other corrections in the Castor eCRFs will only be performed by other authorized research team members (i.e., PhD students, Implementation Lead). In case of corrections, the original data entries will be archived in the system and can be made visible. For all data entries and corrections, the date, time of day and person who is performing the entries will be generated automatically. This will ensure that any authorized person who may perform data entries and changes in the eCRF can be identified.

7.3 Confidentiality and coding

- Study data will be handled with uttermost discretion and is only accessible to authorized personnel who require the data to fulfil their duties within the scope of the research project. On the eCRFs, participants are only identified by a unique participant code. These ID codes will protect the identities (first and last name) of subjects and will also be treated confidentially.
- The participant identification list (Master file) will be securely stored in a locked cabinet in the Institute of Nursing Science, separate from the project data. Only two authorized members (i.e., the Implementation Lead and one researcher) of the research team will have access to the cabinet. All identifiers will be removed during the transcription of interviews (sample 1C). Researchers working in data analysis will only obtain pseudonymized IDs of participants.
- Recruitment feasibility data and downloaded NoMAD results from Limesurvey will be securely stored on the research drive of the Institute of Nursing Science
- Audio recordings will also be stored in a locked cabinet in the Institute of Nursing Science.
- Any paper-based material, including notes from the interviews and meeting logs, and paper-based NoMAD surveys (if applicable), will be stored in the archive in a locked room and cabinet in the Institute of Nursing Science at the University of Basel for 10 years. These facilities can only be accessed by special keys to whom only two people, including the principal investigator and one researcher, have access.
- To protect confidentiality of NoMAD survey participants, any timestamps and identifiers in the LimeSurvey results database will be removed and the results will not be downloaded until the end of the data protection period. Paper-based surveys will be mailed back to the research team at the Institute of Nursing Science.

7.4 Retention and destruction of study data and biological material

All data collected in this project will be archived following strictly the current Swiss legal requirements for data protection and will be performed according to the Ordinance HRO Art. 5 (2). Identifying health related data are stored for 10 years after publication of the research project. The delinked study data may be stored longer to answer new research questions, such as in the case of comparative research with national and international groups. The audio recordings will be will be saved for a period of 10 years and destroyed thereafter. At the end of the project, anonymous INSPIRE feasibility study data will be deposited in an appropriate data depository (e.g., Zenodo), as per funding requirements.
There will be no biological material collected.

8 FUNDING / PUBLICATION / DECLARATION OF INTEREST

This project is funded by Velux Stiftung, the Swiss National Science Foundation, Marie-Curie European Training Network, SSPH+ Global PhD Program in Public Health Sciences (Global P3HS), and the Amt für Gesundheit, Kanton Basel-Landschaft.

Publication Policy:

Publications will include scientific publications. Results will be published in high impact scientific journals in the field of geriatrics, health services research and implementation science and should grant the opportunity for presentations in scientific conferences nationally and internationally. All scientific papers will be original research and will follow green or gold open access policy to support wide distribution.

Conflicts of interests: No conflicts of interest to disclose.

9 REFERENCES

1. The Swiss Federal Council. Ordinance on Clinical Trials in Human Research (HRA) [Internet]. 2013 [cited 2020 Feb 26]. Available from: https://www.admin.ch/opc/en/classified-compilation/20121176/201401010000/810.305.pdf

2. The Swiss Federal Council. CC 810.301 Ordinance of 20 September 2013 on Human Research with the Exception of Clinical Trials (Human Research Ordinance, HRO) [Internet]. [cited 2020 Feb 26]. Available from: https://www.admin.ch/opc/en/classified-compilation/20121177/index.html

3. Europäische Kommission, Europäische Kommission, editors. Adequate social protection for long-term care needs in an ageing society: report jointly prepared by the Social Protection Committee and the European Commission. Luxembourg: Publications Office of the European Union; 2014.

4. Bundesamt für Statistik SE. Alter, Zivilstand, Staatsangehörigkeit [Internet]. 2018, 2020 [cited 2021 June 30]. Available from: https://www.bfs.admin.ch/bfs/de/home/statistiken/bevoelkerung/stand-entwicklung/alter-zivilstand-staatsangehoerigkeit.html

5. Swiss confederation - Federal Statistical Office. Active ageing - Demos 1/2018 [Internet]. 2018 [cited 2018 Aug 13]. Available from: https://www.bfs.admin.ch/bfs/en/home/statistics/catalogues-databases/publications.assetdetail.5046992.html

6. Rechel B, Grundy E, Robine J-M, Cylus J, Mackenbach JP, Knai C, et al. Ageing in the European Union. The Lancet. 2013 Apr 13;381(9874):1312–22.

7. Donovan NJ, Blazer D. Social Isolation and Loneliness in Older Adults: Review and Commentary of a National Academies Report. Am J Geriatr Psychiatry. 2020 Dec;28(12):1233-1244. doi: 10.1016/j.jagp.2020.08.005. Epub 2020 Aug 19. PMID: 32919873; PMCID: PMC7437541.

8. Wiles JL, Leibing A, Guberman N, Reeve J, Allen RES. The Meaning of "Aging in Place" to Older People. The Gerontologist. 2012 Jun 1;52(3):357–66.

9. Davey JA. Accommodation Options for Older People in Aotearoa/New Zealand. Centre for Housing Research Aotearoa New Zealand; 2004. 202 p.

10. Fuller-Thomson E, Yu B, Nuru-Jeter A, Guralnik JM, Minkler M. Basic ADL Disability and Functional Limitation Rates Among Older Americans From 2000–2005: The End of the Decline? J Gerontol A Biol Sci Med Sci. 2009 Dec;64A(12):1333–6.

11. World Health Organization, editor. Global age-friendly cities: a guide. Geneva: World Health Organization; 2007. 76 p.

12. Abdi S, Spann A, Borilovic J, de Witte L, Hawley M. Understanding the care and support needs of older people: a scoping review and categorisation using the WHO international classification of functioning, disability and health framework (ICF). BMC Geriatr. 2019 Jul 22;19(1):195. doi: 10.1186/s12877-019-1189-9. Erratum in: BMC Geriatr. 2020 Jan 22;20(1):23. PMID: 31331279; PMCID: PMC6647108.

13. WHO Regional Office for Europe. Strengthening people-centred health systems in the WHO European Region: framework for action on integrated health services delivery. In Copenhagen, Denmark; 2016.

14. Parry C, Min S-J, Chugh A, Chalmers S, Coleman EA. Further application of the care transitions intervention: results of a randomized controlled trial conducted in a fee-for-service setting. Home Health Care Serv Q. 2009;28(2–3):84–99.

15. World Health Organization, Health Services Delivery Programme, Division of Health Systems and Public Health. Integrated care models: an overview. World Health Organization; 2016.

16. Ham C. Integrated care: What is it? Does it work? What does it mean for the NHS? Integr Care. 2011;8.

17. Craig P, Dieppe P, Macintyre S, Michie S, Nazareth I, Petticrew M. Developing and evaluating complex interventions: the new Medical Research Council guidance. BMJ [Internet]. 2008 Sep 29 [cited 2020 Feb 26];337. Available from: https://www.bmj.com/content/337/bmj.a1655

18. Leijten FRM, Struckmann V, van Ginneken E, Czypionka T, Kraus M, Reiss M, et al. The SELFIE framework for integrated care for multi-morbidity: Development and description. Health Policy. 2018 Jan;122(1):12–22.

19. World Health Organization. Key components of a well functioning health system [Internet]. 2010 [cited 2020 Feb 26]. Available from: https://www.who.int/healthsystems/EN_HSSkeycomponents.pdf?ua=1

20. Deschodt M, Laurent G, Cornelissen L, Yip O, Zúñiga F, Denhaerynck K, et al. Core components and impact of nurse-led integrated care models for home-dwelling older people: A systematic review and meta-analysis. Int J Nurs Stud. 2020 Feb 29;105:103552.

21. Hopman P, de Bruin SR, Forjaz MJ, Rodriguez-Blazquez C, Tonnara G, Lemmens LC, et al. Effectiveness of comprehensive care programs for patients with multiple chronic conditions or frailty: A systematic literature review. Health Policy, 2016;120(7):818-32. doi:10.1016/j.healthpol.2016.04.002

22. Looman WM, Huijsman R, Fabbricotti IN. The (cost-)effectiveness of preventive, integrated care for community-dwelling frail older people: A systematic review. Health and Social Care in the Community, 2019;27(1):1-30. doi:10.1111/hsc.12571

23. Yip O, Huber E, Stenz S, Zullig LL, Zeller A, De Geest SM, et al.. A Contextual Analysis and Logic Model for Integrated Care for Frail Older Adults Living at Home: The INSPIRE Project. International Journal of Integrated Care. 2021;21(2):9. DOI: http://doi.org/10.5334/ijic.5607

24. Smit LC, Schuurmans MJ, Blom JW, Fabbricotti IN, Jansen APD, Kempen G, et al. Unravelling complex primary-care programs to maintain independent living in older people: a systematic overview. *Journal of Clinical Epidemiology*, 2018; 96: 110–9. DOI: https://doi.org/10.1016/j.jclinepi.2017.12.013

25. Eccles MP, Mittman BS. Welcome to implementation science. ImplementationSci. 2006;1:1. doi: 10.1186/1748-5908-1-1

26. Peters DH, Adam T, Alonge O, Agyepong IA, Tran N. Implementation research: what it is and how to do it. *BMJ*, 2013; 347. DOI: https://doi.org/10.1136/bmj.f6753

27. Neta G, Brownson RC, Chambers DA. Opportunities for epidemiologists in implementation science: A primer. Am J Epidemiol. 2018;187(5):899–910. doi:. http://dx.doi.org/10.1093/aje/kwx323 PubMed

28. Federal Statistical Office. Switzerland's population 2019 | Publication [Internet]. Federal Statistical Office. 2019 [cited 2021 Jun 30]. Available from: /content/bfs/en/home/statistiken/bevoelkerung.assetdetail.10827609.html

29. World Health Organization. World report on ageing and health. World Health Organization; 2015. [cited 15 Dec 2018]. Available from: https://www.who.int/ageing/events/world-report-2015-launch/en/

30. Stewart AL, Nápoles AM, Piawah S, Santoyo-Olsson J, Teresi JA. Guidelines for Evaluating the Feasibility of Recruitment in Pilot Studies of Diverse Populations: An Overlooked but Important Component. *Ethn Dis*. 2020;30(Suppl 2):745-754. Published 2020 Nov 19. doi:10.18865/ed.30.S2.745

31. Proctor E, Silmere H, Raghavan R, Hovmand P, Aarons G, Bunger A, et al. Outcomes for Implementation Research: Conceptual Distinctions, Measurement Challenges, and Research Agenda. Adm Policy Ment Health Ment Health Serv Res. 2011 Mar 1;38(2):65–76.

32. Finch, T.L., Girling, M., May, C.R. *et al.* Improving the normalization of complex interventions: part 2 - validation of the NoMAD instrument for assessing implementation work based on normalization process theory (NPT). *BMC Med Res Methodol* **18,** 135 (2018). https://doi.org/10.1186/s12874-018-0591-x

33. Steverink N, Slaets JPJ, Schuurmans H, van Lis M. Measuring frailty: Developing and testing the GFI (Groningen frailty indicator). Gerontologist. 2001 Oct;41:236–7.

34. Peters LL, Boter H, Buskens E, Slaets JPJ. Measurement Properties of the Groningen Frailty Indicator in Home-Dwelling and Institutionalized Elderly People. J Am Med Dir Assoc. 2012 Jul;13(6):546–51.

35. Integrated care for older people (ICOPE): Guidance for person-centred assessment and pathways in primary care. Geneva: World Health Organization; 2019 (WHO/FWC/ALC/19.1). Licence: CC BY-NC-SA 3.0 I

36. Borson. Mini-Cog. Instructions for Administration & Scoring. Available from: https://mini-cog.com/mini-cog-instrument/scoring-the-mini-cog/

37. Tsoi KK, Chan JY, Hirai HW, Wong SY, Kwok TC. Cognitive Tests to Detect Dementia: A Systematic Review and Meta-analysis. JAMA Intern Med. 2015 Sep;175(9):1450-8. doi: 10.1001/jamainternmed.2015.2152. PMID: 26052687.

38. Rubenstein LZ, Harker JO, Salvà A, Guigoz Y, Vellas B. Screening for undernutrition in geriatric practice: developing the short-form mini-nutritional assessment (MNA-SF). J Gerontol A Biol Sci Med Sci. 2001 Jun;56(6):M366-372.

39. Lilamand, M., Kelaiditi, E., Cesari, M. *et al.* Validation of the Mini Nutritional Assessment-Short Form in a population of frail elders without disability. Analysis of the Toulouse Frailty Platform population in 2013. *J Nutr Health Aging* **19,** 570–574 (2015). https://doi.org/10.1007/s12603-015-0457-4

40. Panel on Prevention of Falls in Older Persons, American Geriatrics Society and British Geriatrics Society. Summary of the updated American Geriatrics Society/British Geriatrics Society clinical practice guideline for prevention of falls in older persons. *J Am Geriatr Soc.* 2011; 59(1): 148–157.

41. Malterud, K., Siersma, V. D., & Guassora, A. D. (2016). Sample size in qualitative interview studies: guided by information power. Qualitative health research, 26(13), 1753-1760.

42. Hamilton AB. 2013. Rapid Qualitative Methods in Health Services Research: Spotlight on Women's Health VA HSR&D National Cyberseminar series: Spotlight on Women's Health; December 2013

43. World Medical Association. Declaration of Helsinki – Ethical Principles for Medical Research Involving Human Subjects [Internet]. [cited 2020 Feb 26]. Available from: https://www.wma.net/policies-post/wma-declaration-of-helsinki-ethical-principles-for-medical-research-involving-human-subjects/
